# Hepcidin contributes to Swedish mutant APP-induced osteoclastogenesis and trabecular bone loss

**DOI:** 10.1038/s41413-021-00146-0

**Published:** 2021-06-09

**Authors:** Hao-Han Guo, Lei Xiong, Jin-Xiu Pan, Daehoon Lee, Kevin Liu, Xiao Ren, Bo Wang, Xiao Yang, Shun Cui, Lin Mei, Wen-Cheng Xiong

**Affiliations:** 1 Department of Neurosciences, School of Medicine, Case Western Reserve University, Cleveland, OH USA; 2 Louis Stokes Cleveland Veterans Affairs Medical Center, Cleveland, OH USA; 3 Department of Neuroscience and Regenerative Medicine, Medical College of Georgia, Augusta University, Augusta, GA USA

**Keywords:** Osteoporosis, Bone

## Abstract

Patients with Alzheimer’s disease (AD) often have lower bone mass than healthy individuals. However, the mechanisms underlying this change remain elusive. Previously, we found that Tg2576 mice, an AD animal model that ubiquitously expresses Swedish mutant amyloid precursor protein (APP_swe_), shows osteoporotic changes, reduced bone formation, and increased bone resorption. To understand how bone deficits develop in Tg2576 mice, we used a multiplex antibody array to screen for serum proteins that are altered in Tg2576 mice and identified hepcidin, a master regulator of iron homeostasis. We further investigated hepcidin’s function in bone homeostasis and found that hepcidin levels were increased not only in the serum but also in the liver, muscle, and osteoblast (OB) lineage cells in Tg2576 mice at both the mRNA and protein levels. We then generated mice selectively expressing hepcidin in hepatocytes or OB lineage cells, which showed trabecular bone loss and increased osteoclast (OC)-mediated bone resorption. Further cell studies suggested that hepcidin increased OC precursor proliferation and differentiation by downregulating ferroportin (FPN) expression and increasing intracellular iron levels. In OB lineage cells, APP_swe_ enhanced hepcidin expression by inducing ER stress and increasing OC formation, in part through hepcidin. Together, these results suggest that increased hepcidin expression in hepatocytes and OB lineage cells in Tg2576 mice contributes to enhanced osteoclastogenesis and trabecular bone loss, identifying the hepcidin-FPN-iron axis as a potential therapeutic target to prevent AD-associated bone loss.

## Introduction

Alzheimer’s disease (AD) is the most common form of dementia. Patients with AD suffer from a decline in memory, an inability to recall the recent past and eventual loss of long-term memories, loss of cognitive function, and changes in personality. Interestingly, patients with AD often have lower bone mineral density (BMD) and a higher rate of hip fracture, which are features of osteoporosis, than healthy individuals,^1,2^ raising the question of how these disorders are linked. Although AD is believed to be a neurodegenerative disease and osteoporosis results from decreased osteoblast (OB)-mediated bone formation and/or increased osteoclast (OC)-mediated bone resorption, both AD and osteoporosis share similar risk factors.^2^ Both are age-associated degenerative disorders;^2,3^ both occur more frequently in postmenopausal women,^4,5^ and both are associated with genetic and environmental factors, such as oxidative stress^6–9^ and chronic inflammation.^10,11^ While numerous proinflammatory cytokines are believed to contribute to the pathogenesis of both diseases,^12–14^ the mechanisms underlying the association between AD and osteoporosis remain elusive.

App (amyloid precursor protein) is a gene associated with early-onset AD that is inherited in a Mendelian ratio. Mutations in APP (e.g., Swedish mutation, APPswe) favor APP cleavage and thus the generation of Aβ_40-42_. Therefore, much research on AD focused on the accumulation of Aβ_40-42_ in the brain, even though it is widely recognized that App and other AD risk genes are expressed not only in the brain but also in peripheral tissues, including bone cells. We examined APPswe’s contribution to AD-associated bone loss through the use of two animal models, Tg2576 and TgAPPswe-Ocn mice. Tg2576 mouse model is a well-characterized AD animal model that ubiquitously expresses APPswe. Interestingly, this model displays early-onset osteoporotic changes months before any pathological defects in the brain can be detected.^15,16^ The TgAPPswe-Ocn model is a conditional transgenic mouse model that expresses APPswe in an osteocalcin (Ocn)-Cre-dependent manner and thus selectively expresses APPswe in OB lineage cells.^9,16,17^ This model recapitulates the osteoporotic changes observed in the *Tg2576* mouse model, suggesting that APPswe plays a cell-autonomous role in the suppression of bone formation and bone mass homeostasis.^9,16^

To further understand how APPswe regulates bone homeostasis, we identified hepcidin as a potential downstream factor of APPswe. Hepcidin, which is encoded by the hamp1 gene in mice, is a peptide hormone released mainly by liver hepatocytes.^18,19^ It acts as a key regulator of systematic iron homeostasis by binding of its N-terminus to ferroportin (FPN), the only known iron exporter that is largely expressed in macrophages and intestinal cells.^20–22^ Upon hepcidin binding, FPN is internalized and degraded, leading to a decrease in the export of intracellular iron from macrophages and intestinal cells and thus reducing serum but increasing intracellular iron levels.^20^ Hepcidin expression in hepatocytes can be induced by multiple factors, including proinflammatory cytokines,^22–28^ iron overload,^19,29^ bone morphogenetic protein (BMP) 6,^30,31^ and endoplasmic reticulum (ER) stress.^32,33^ Interestingly, many of these hepcidin regulators are also implicated in the pathogenesis of both AD and osteoporosis. Recent studies have suggested that hepcidin and iron metabolism are involved in osteoporosis. Hepcidin treatment increases intracellular iron and promotes osteoclast differentiation of RAW264.7 cells.^34^ Iron overload, which is coupled with overexpression of hepcidin by the liver, contributes to unloading-induced bone loss.^35^ Studies have also shown that FPN in myeloid osteoclast precursors has an important role in regulating intracellular iron levels, osteoclastogenesis, and skeletal homeostasis in mice.^36^ However, little is known regarding the contribution of hepcidin to AD or AD-associated osteoporosis.

Here, we provide evidence that hepcidin is induced by APPswe-driven ER stress and that increased hepcidin expression contributes to trabecular bone loss. Hepcidin levels are elevated not only in the serum but also in the liver, muscle, and OB lineage cells of young adult Tg2576 mice. Overexpression of hepcidin in hepatocytes or OB lineage cells results in a loss of trabecular bone mass in young adult mice. Such bone loss deficits appear to be due in large part to increases in osteoclastogenesis and OC-mediated bone resorption, although a decrease in bone formation is detected in mice expressing hepcidin in OB lineage cells but not in hepatocytes. Cell studies not only confirmed the function of hepcidin in promoting OC differentiation but also revealed an unrecognized role of hepcidin in increasing the proliferation of OC precursors. These cellular functions are likely due to hepcidin-induced downregulation of FPN expression and increased intracellular iron levels in OC precursors. Moreover, APPswe in OB lineage cells increases hepcidin expression, likely by ER stress, and promotes OC formation in part by OB-derived hepcidin. In summary, these results demonstrate that hepcidin expressed in hepatocytes and OB lineage cells is a critical regulator of osteoclastogenesis, revealing a link between the liver and bone and identifying the hepcidin-FPN-iron pathway as a therapeutic target for AD-associated osteoporosis.

## Results

### Increased hepcidin levels in young adult Tg2576 and aged WT mice

**Fig. 1:**
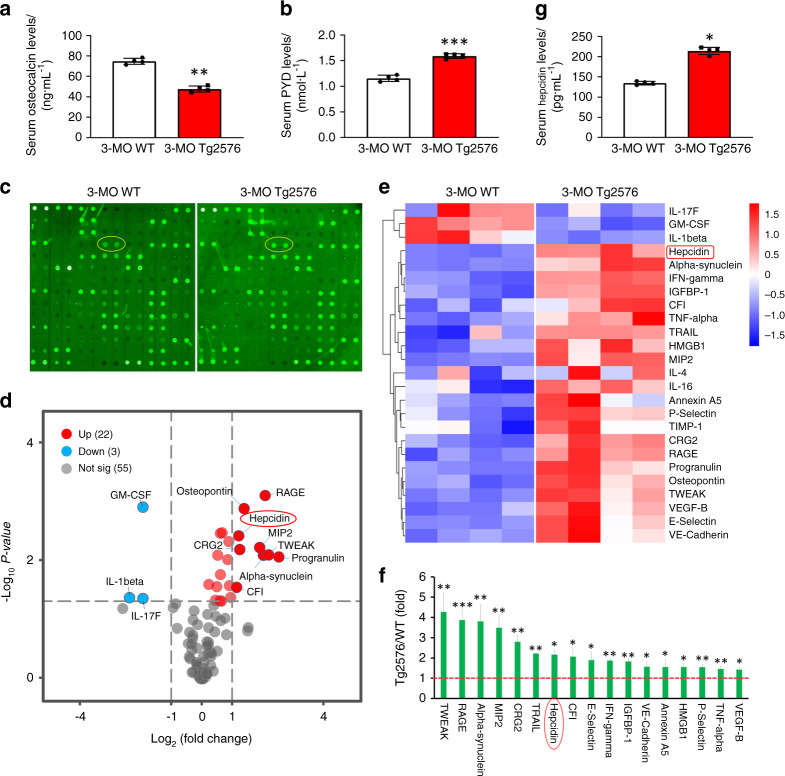
Increased expression levels of hepcidin in serum samples from 3-month-old Tg2576 mice. **a** Serum osteocalcin (Ocn) levels in 3-month-old WT and Tg2576 mice. Serum osteocalcin levels were measured by ELISA. The mean ± SD of measurements from four males per genotype are shown. **P < 0.01 (significant difference). **b** ELISA analysis of serum pyridinoline (PYD) levels. The mean ± SD (*n* = 4) are presented. ***P < 0.001 (significant difference). **c** Proteome profile of the sera of 3-month-old WT and Tg2576 mice, as determined by a mouse cytokine array. Hepcidin is marked with a yellow circle. **d** Volcano plot of the Tg2576/WT Log_2_ ratio (fold change). The upregulated proteins are marked in red, and the downregulated proteins are indicated in blue (*P* < 0.05). **e** Heat map of the protein expression z-scores computed for the 25 proteins that are differentially expressed in the sera of 3-month-old Tg2576 mice (*P* < 0.05). **f** The fold change in the expression of 17 proteins in the sera of 3-month-old Tg2576 mice. These proteins also showed the same changes in 7-month-old Tg2576 mice. *P < 0.05; **P < 0.01; ***P < 0.001 (significant difference). **g** Analysis of serum hepcidin levels in 3-month-old WT and Tg2576 mice by ELISA. *P < 0.05 (significant difference)

To investigate the mechanisms underlying AD-associated bone loss, we examined bone phenotypes in the Tg2576 mouse model, a well-characterized AD mouse model that expresses APPswe under the control of the Prion promoter.^15,37^ Tg2576 mice show osteoporotic changes in young adulthood,^15^ which is likely due to a decrease in OB-mediated bone formation and age-dependent increase in OC-mediated bone resorption.^15,16^ To further understand how osteoporotic changes are induced in Tg2576 mice, we screened for potential protein(s) that may regulate OB or OC function in serum samples from Tg2576 mice through the use of a multiplexed antibody array. Serum samples from 3-month-old Tg2576 mice were initially examined, and a decrease in the level of osteocalcin (a bone formation marker) and an increase in the level of PYD (deoxypyridinoline) (a bone resorption marker) were detected in serum samples from Tg2576 mice compared with samples from WT controls at this age (Fig. 1a, b). Nitrocellulose membranes containing 90 antibodies against secreted proteins that are critical for inflammation, angiogenesis, and cell growth were incubated with biotinylated serum protein samples from Tg2576 and WT controls (Supplementary Table S1). Proteins bound to the antibodies on the nitrocellulose membrane were detected using streptavidin-conjugated fluorescent reagents as described in the Materials and Methods section. Approximately 25 proteins showed significant changes in serum samples from Tg2576 mice compared with those from age-matched WT controls (Fig. 1c–f). Among these 25 proteins, we focused on hepcidin because it is a key regulator of ion homeostasis, which is altered in both AD and osteoporosis,^38,39^ but its function in both diseases remains elusive.

The increase in hepcidin expression in serum samples from 3-month-old Tg2576 mice was further confirmed by ELISA (Fig. 1g). We then asked whether the increases in serum hepcidin levels are due to increased hepcidin expression. To this end, the expression of hamp1 (the gene encoding hepcidin) in various tissues from Tg2576 mice (3-month-old) was measured by RT-PCR and ELISA. hamp1 mRNA levels in the liver, muscle, and OB lineage cells (BMSCs), but not in the cortex, hippocampus, or OC lineage cells (BMMs), were significantly increased in Tg2576 mice compared to WT control mice (Supplementary Fig. S1a). Consistent with these results, hepcidin protein levels (measured by ELISA) in the liver, muscle, and OB lineage cells (BMSCs), but not the hippocampus or BMMs, were higher in Tg2576 mice than in controls (Supplementary Fig. S1b). Notably, among the different tissues, the liver contained the highest level of hamp1, which is in line with previous reports.^18,19^ OB lineage cells (BMSCs) also expressed higher levels of hamp1 than OC lineage cells (BMMs) (Supplementary Fig. S1), and ELISA but not RT-PCR revealed increased hepcidin levels in the cortices of Tg2576 mice (Supplementary Fig. S1). These results suggest that the increased serum hepcidin levels in Tg2576 mice are largely due to increased hamp1 expression in various tissues, including the liver, muscles, and OB lineage cells.

Given that aging is a risk factor for both AD and osteoporosis,^2,3,8,40^ we measured serum levels of hepcidin in various aged WT mice by ELISA. Indeed, hepcidin serum levels were increased in an age-dependent manner, with a significant increase starting at 18 months of age in mice (Supplementary Fig. S2a). As hepcidin is a key factor for systemic iron homeostasis, we examined serum levels of ferrous iron (Fe^2+^) and total iron (Fe^2+^ and Fe^3+^) in various aged mice. The serum levels of Fe^2+^ and total iron (Fe^2+^ and Fe^3+^) were also increased in mice aged 18 months and older (Supplementary Fig. S2b, c). Notably, the serum levels of the bone formation marker osteocalcin were reduced (Supplementary Fig. S2d), but the serum levels of the bone resorption marker PYD were elevated in an age-dependent manner (Supplementary Fig. S2e), which is in line with previous reports.^41^ These results suggest an age-dependent association of serum hepcidin levels with a decrease in bone formation and/or an increase in bone resorption. Together, these results demonstrate that hepcidin levels are increased not only in serum samples from young adult Tg2576 mice but also in serum samples from aged WT mice, implicating hepcidin in the pathogenesis of aged-associated AD and osteoporosis.

### Trabecular bone loss in mice expressing hepcidin in hepatocytes or OB lineage cells

**Fig. 2:**
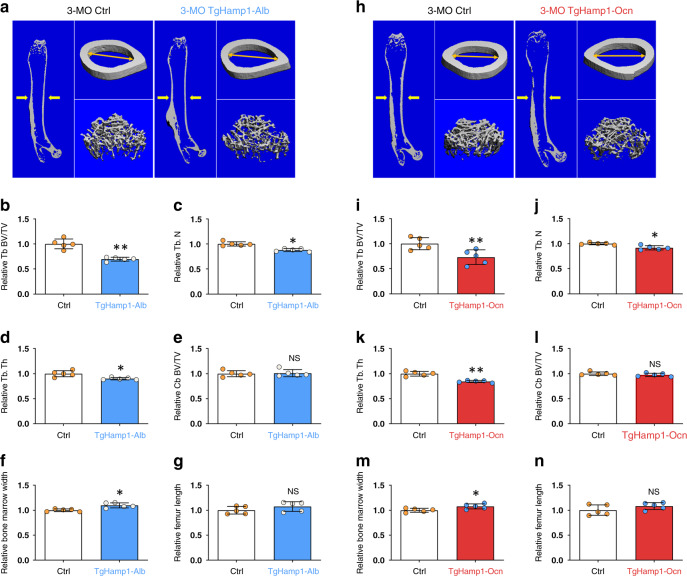
Decreased trabecular bone mass in 3-month-old TgHamp1-Alb and TgHamp1-Ocn mice. **a** μCT analysis of femurs from 3-MO Ctrl and TgHamp1-Alb littermates. Five different male mice of each genotype were examined blindly. Representative 3D images are shown in (**a**). **b**–**g** Quantitative analyses of the trabecular bone (Tb) volume over total volume (BV/TV), Tb number (Tb. N), Tb thickness (Tb. Th), cortical bone (Cb) volume/TV, bone marrow width and femur length by direct μCT. The data are in (**b**–**g**) are presented as the mean ± SD. *P < 0.05; **P < 0.01; NS, no significant difference. **h** μCT analysis of femurs from 3-month-old Ctrl and TgHamp1-Ocn littermates. Five different male mice of each genotype were examined blindly. Representative 3D images are shown in (**h**). **i**–**n** Quantitative analyses of Tb BV/TV, Tb. N, Tb. Th, Cb BV/TV, bone marrow width and femur length by direct μCT. The data in (**i**–**n**) are presented as the mean ± SD. *P < 0.05; **P < 0.01; NS no significant difference

Given that increased Hamp1 expression was observed in the liver and OB lineage cells in Tg2576 mice, we generated two transgenic mouse lines, TgHamp1-Alb and TgHamp1-Ocn, to selectively express hepcidin in liver hepatocytes and OB lineage cells, respectively. TgHamp1-Alb and TgHamp1-Ocn mice were generated by crossing newly generated LSL-Hamp1 (TgHamp1) mice with albumin (Alb) promotor-driven Cre (Alb-Cre) and osteocalcin promotor-driven Cre (Ocn-Cre) mice, respectively (Supplementary Fig. S3a). In LSL-Hamp1 mice, Hamp1 mRNA expression is under the control of the chicken β-actin promoter (CAGGS) with a CMV enhancer, but Hamp1 protein expression depends on the excision of the STOP signal by Cre (Supplementary Fig. S3a). In Alb-Cre mice, Cre is known to be selectively expressed in liver hepatocytes,^42^ and in Ocn-Cre mice, Cre is expressed largely in OB lineage cells.^17,43^ The body sizes and body weights of 3-month-old TgHamp1-Alb and TgHamp1-Ocn mice appeared to be comparable to those of age- and sex-matched controls (Supplementary Fig. S3b–f). As expected, TgHamp1-Alb and TgHamp1-Ocn mice showed elevated hepcidin levels in liver and OB lineage cells, respectively (Supplementary Fig. S3g–j). In addition, hepcidin levels in the circulation (serum) were elevated, and the serum levels of iron (Fe^2+^ and Fe^3+^) were decreased in both TgHamp1-Alb and TgHamp1-Ocn mice compared with littermate controls (Supplementary Fig. S3k–n). We then assessed long bone (femur) mass in both TgHamp1-Alb and TgHamp1-Ocn mice and controls by microcomputer tomographic (μCT) analysis. At 3 months of age, both TgHamp1-Alb and TgHamp1-Ocn mice showed lower trabecular bone mass but not cortical bone mass than control mice (Fig. 2a, b, e, h, i and l). Although there was no significant difference in femur length (Fig. 2g, n), decreases in trabecular bone volume/total volume (Tb. BV/TV), trabecular bone number (Tb. N), and trabecular thickness (Tb. Th) and increases in bone marrow width were detected in both TgHamp1-Alb and TgHamp1-Ocn mice (Fig. 2b–g, i–n). These trabecular bone changes were confirmed by H&E staining analysis in 3-month-old TgHamp1-Alb (Supplementary Fig. S4f–j) and TgHamp1-Ocn mice (Supplementary Fig. S5f–j) but undetectable in 1-month-old mutant mice (Supplementary Fig. S4a-e, Supplementary Fig. S5a–e). These osteoporotic changes were detected in both male but female TgHamp1-Alb and TgHamp1-Ocn mice (Supplementary Fig. S4k–o, Supplementary Fig. S5k–o). Together, these results suggest that hepcidin derived from either hepatocytes or OB lineage cells plays a negative role in maintaining trabecular bone mass.

### Impaired OB-mediated bone formation in TgHamp1-Ocn, but not TgHamp1-Alb, mice

Bone mass is determined by OB-mediated bone formation and OC-mediated bone resorption. We thus examined both processes in TgHamp1-Alb, TgHamp1-Ocn, and control mice. Bone formation was first evaluated by injecting fluorochrome-labeled calcein green into 3-month-old TgHamp1-Alb, TgHamp1-Ocn, and control littermates twice at a 10-d interval. The mineral apposition rate (MAR), mineral surface/bone surface (MS/BS), and bone formation rate (BFR) in both the endocortical (Ec.) and trabecular (Tb.) bone regions were analyzed in nondecalcified femur sections from the injected mice. To our surprise, no significant difference was observed in Ec.MAR, Tb.MAR, Ec.MS/BS, Tb.MS/BS, Ec.BFR or Tb.BFR between TgHamp1-Alb and control mice (Supplementary Fig. S6a–d), suggesting normal bone formation. This finding was further confirmed by analysis of serum levels of osteocalcin (Supplementary Fig. S6e), a bone formation marker, and in vitro osteoblastogenesis by ELISA (Supplementary Fig. S7a–c). The mRNA levels of osteoblastogenesis-related genes, including runt-related transcription factor 2 (Runx2), transcription factor Sp7 (Sp7), alpha-1 type I collagen (Col1α1) and osteopontin (Opn), were not different between OB progenitors derived from TgHamp1-Alb mice compared to those derived from control mice (Supplementary Fig. S7d). In addition, there were no significant differences in proliferation and apoptosis between BMSCs derived from TgHamp1-Alb mice and those derived from control mice (Supplementary Fig. S8a, b). The Runx2^+^ cell number in trabecular bone and cortical bone regions was also not different between 3-month-old TgHamp1-Alb mice and control mice (Supplementary Fig. S9a, b). These results suggest that hepatocyte-derived hepcidin has little or no effect on OB-mediated bone formation.

Unlike in TgHamp1-Alb mice, the MAR, MS/BS, and BFR of trabecular bone but not Ec. cortical bone were decreased (Supplementary Fig. S6f–i), indicating a decrease in trabecular bone formation. This finding was further supported by decreases in serum levels of the bone formation marker osteocalcin (Supplementary Fig. S6j) and the proliferation, differentiation, and function of OB progenitors derived from TgHamp1-Ocn mice (Supplementary Fig. S7c, d, S8c, d, and S9c, d). Together, these results suggest that OB-derived hepcidin, unlike hepatocyte-derived hepcidin, inhibits OB-mediated bone formation in trabecular, but not cortical, bones.

### Increased OC-mediated bone resorption in both TgHamp1-Alb and TgHamp1-Ocn mice

**Fig. 3:**
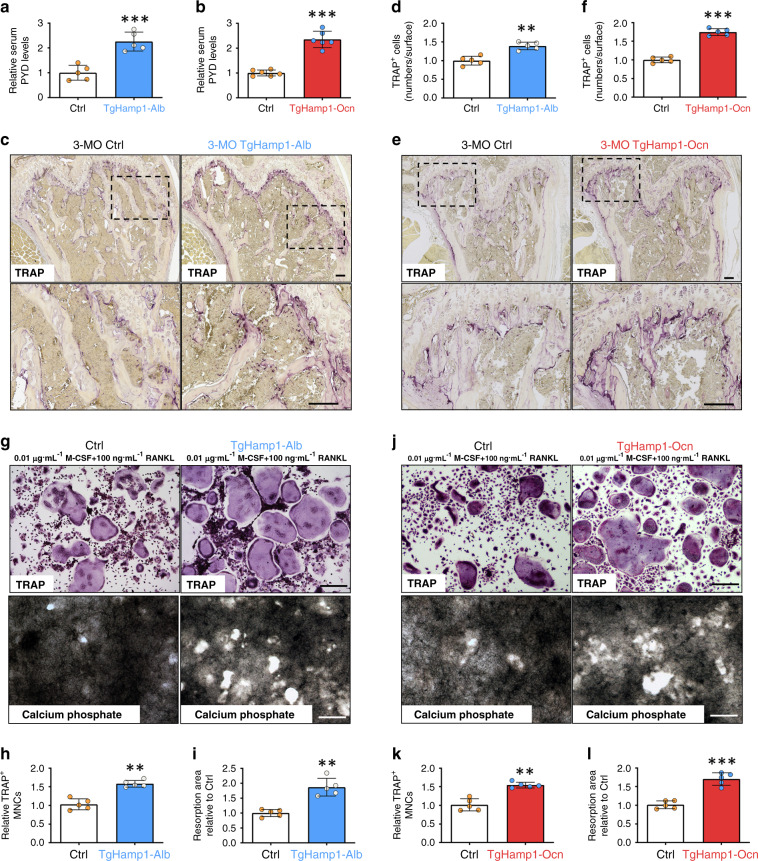
Increased bone resorption in 3-month-old TgHamp1-Alb and TgHamp1-Ocn mice. **a**, **b** Analysis of serum PYD levels in 3-month-old male Ctrl, TgHamp1-Alb and TgHamp1-Ocn littermates by ELISA. The mean ± SD of measurements from five different mice of each genotype and relative to the Ctrl group are shown. ***P < 0.001; NS, no significant difference. **c**, **e** TRAP staining analysis of femur sections from 3-month-old Ctrl, TgHamp1-Alb and TgHamp1-Ocn mice. Bar, 150 μm. **d**, **f** The values are shown as the mean ± SD of measurements from five different male mice of each genotype. **P < 0.01; ***P < 0.001; NS, no significant difference. **g**, **j** TRAP staining and bone resorption pit analysis of cultured OCs derived from BMMs from mice of different genotypes on day 7. Cells were treated with 0.01 μg·mL^−1^ M-CSF and 100 ng·mL^−1^ RANKL for 7 d. Bar, 200 μm. For the bone resorption pit assay, OCs were cultured in plates coated with calcium phosphate matrix. **h**, **k** The number of TRAP^+^ multinucleated cells (MNCs; more than three nuclei) per randomly selected visual field is shown in (**g**) and (**j**). The data are presented as the mean ± SD of five or six different cell culture experiments. **P < 0.01. **i**, **l** Resorptive activity was quantified based on the mean resorption area in (**g**) and (**j**). The data are presented as the mean ± SD of five different cell culture experiments. **P < 0.01

We next evaluate bone resorption in TgHamp1-Alb, TgHamp1-Ocn, and control mice. Bone resorption was first evaluated by measuring the serum levels of the bone resorption marker PYD, which showed a marked increase in both TgHamp1-Alb and TgHamp1-Ocn mice compared with control mice (Fig. 3a, b). We then asked whether this increased bone resorption is caused by enhanced osteoclastogenesis in the mutant mice. Indeed, bone histomorphological examinations showed a significant increase in the number of TRAP^+^ OCs per unit of bone surface in femurs, particularly in the trabecular bone regions, in both TgHamp1-Alb and TgHamp1-Ocn mice (Fig. 3c–f). Thus, these in vivo results demonstrate that OC formation and activation are elevated in mice expressing hepcidin in hepatocytes or OB lineage cells.

### Hepcidin promotion of the proliferation, differentiation, and activation of cultured OC progenitor cells

**Fig. 4:**
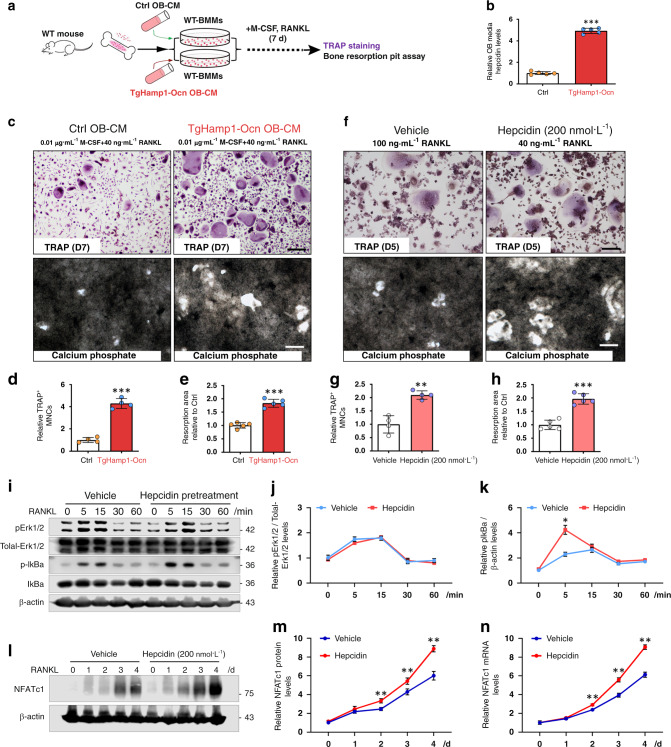
Hepcidin promotes the differentiation and activation of cultured OC progenitor cells. **a** Conditioned medium (CM) treatment strategy. **b** Analysis of hepcidin levels following OB-CM and TgHamp1-Ocn OB-CM treatment by ELISA. CM from five different cell culture experiments for each genotype was used, and the mean ± SD relative to the Ctrl group are shown. ***P < 0.001. **c** TRAP staining and bone resorption pit analysis of cultured OCs derived from wild-type BMMs in the presence of Ctrl OB-CM or TgHamp1-Ocn OB-CM. Cells were treated with 0.01 μg·mL^−1^ M-CSF and 40 ng·mL^−1^ RANKL for 7 d as indicated. Bar, 200 μm. **d**, **e** Quantification of TRAP^+^ MNCs and quantification of resorptive activity in **c**. The data are presented as the mean ± SD of four or five different cell culture experiments. ***P < 0.001. **f** TRAP staining and bone resorption pit analysis of cultured OCs derived from RAW264.7 cells in the presence of vehicle or 200 nmol·L^−1^ hepcidin peptide. Cells were treated with 100 ng·mL^−1^ RANKL for 5 d or 8 d as indicated. Bar, 200 μm. **g**, **h** The number of TRAP^+^ MNCs and quantification of resorptive activity in **f**. The data are presented as the mean ± SD of four or five different culture experiments. **P < 0.01; ***P < 0.001. **i**–**k** RANKL signaling in RAW264.7 cells was assessed by western blot analysis of lysates stimulated with RANKL for the indicated times. RAW264.7 cells were serum-starved overnight and then pretreated with vehicle or hepcidin (200 nmol·L^−1^) for 4 h. The data were quantified by ImageJ software and are presented in (**j**) and (**k**). *P < 0.05. **l**–**n** Protein and mRNA expression of NFATC1 in RAW264.7 cells treated with hepcidin were measured by western blot or real-time PCR. The quantitative data were shown in (**m**) and (**n**). **P < 0.01

We then asked whether hepcidin regulates OC formation and resorptive activity in vitro. To this end, we first evaluated M-CSF- and RANKL-induced OC differentiation of BMMs derived from TgHamp1-Alb, TgHamp1-Ocn, and control mice. Cultured BMMs derived from TgHamp1-Alb and TgHamp1-Ocn mice showed more TRAP^+^ OCs than cultured BMMs derived from control mice on day 5 of RANKL treatment (Fig. 3g, h, j, k), indicating that more OC progenitors were present in the cultured BMMs derived from TgHamp1-Alb TgHamp1-Ocn mice. We next asked whether these OCs are active by examining their resorptive activity in culture. In line with the increased OC formation, hyperresorptive activity and an increase in the mean size of resorptive pits was detected in cultured OCs derived from TgHamp1-Alb and TgHamp1-Ocn mice compared with those derived from control mice (Fig. 3g, i, j, l). We next examined whether conditioned medium (CM) from Hamp1^+^ OB lineage cells can promote OC differentiation and bone resorptive activity. CM from OBs derived from control and TgHamp1-Ocn mice was collected. BMMs from wild-type mice were treated with CM for 7 d in the presence of 0.01 μg·mL^−1^ M-CSF and a lower concentration (40 ng·mL^−1^) of RANKL to induce OC differentiation (Fig. 4a). In the presence of hepcidin^+^ CM (Fig. 4b), the number of TRAP^+^ OCs and the size of resorptive pits were increased (Fig. 4c–e), suggesting that hepcidin plays a positive role in RANKL-induced OC formation and bone resorptive activity. Then, we examined recombinant hepcidin’s effect on OC differentiation and resorptive activity using RAW264.7 cells (a macrophage cell line). Increases in the number of OCs and the size of resorptive pits were detected once RAW264.7 cells were exposed to a recombinant hepcidin peptide (200 ng·mL^−1^) and RANKL (Fig. 4f–h), suggesting that the hepcidin peptide is sufficient to enhance RANKL-induced OC formation.

To further understand how hepcidin promotes RANKL-induced OC formation, we assessed RANKL-induced signaling events in macrophages pretreated with hepcidin. RAW264.7 cells were used because they can survive in the absence of M-CSF. RAW264.7 cells were serum-starved overnight and pretreated with hepcidin (200 nmol·L^−1^) or vehicle for 4 h. RANKL-driven signaling events, including phosphorylation of Erk1/2 and IκBα, were examined, as they are essential for the development of macrophages into mature OCs and for macrophage survival.^44,45^ Transient phosphorylation of Erk1/2 and IκBα was detected 5 and 15 min after RANKL stimulation in both groups (Fig. 4i–k); however, the p-IκBα level increased more after 5 min of stimulation in the hepcidin-pretreated cells than in the vehicle-pretreated cells (Fig. 4k). In addition, both the protein levels and the mRNA levels of NFATC1, a master transcription factor of osteoclastogenesis, were significantly increased in RAW264.7 cells treated with hepcidin (Fig. 4 l–n).

We also examined hepcidin’s role in BMM and RAW264.7 cell proliferation, a crucial initial event for OC development.^46^ RAW264.7 cells were cultured in the presence of CM from control and TgHamp1-Ocn OBs for 4 h. EdU (10 μmol·L^−1^) was added to the cells 1 h before they were fixed for immunostaining analysis. As shown in Supplementary Fig. 10, the EdU^+^ cell density was higher in RAW264.7 cells exposed to hepcidin-containing CM than those exposed to control CM (Supplementary Fig. 10a, b), suggesting that hepcidin increased in RAW264.7 cell proliferation. In line with this finding, increases in the number of Ki67^+^ and pH3^+^ RAW264.7 cells were observed in response to hepcidin-containing CM (Supplementary Fig. 10a, c, d). The hepcidin-induced increase in BMM/RAW264.7 cell proliferation was dose-dependent, with the peak increase being induced by 2 000 nmol·L^−1^ hepcidin peptide (Supplementary Fig. S10e–j). These results suggest that both secretory hepcidin in CM and recombinant hepcidin peptide can promote differentiation and proliferation of BMMs and RAW264.7 cells, revealing the cellular mechanism underlying the hepcidin-induced increase in the number of OC progenitors and/or osteoclastogenesis.

### Downregulation of FPN expression, the mechanism underlying the hepcidin-induced increase in macrophage proliferation and OC formation

**Fig. 5:**
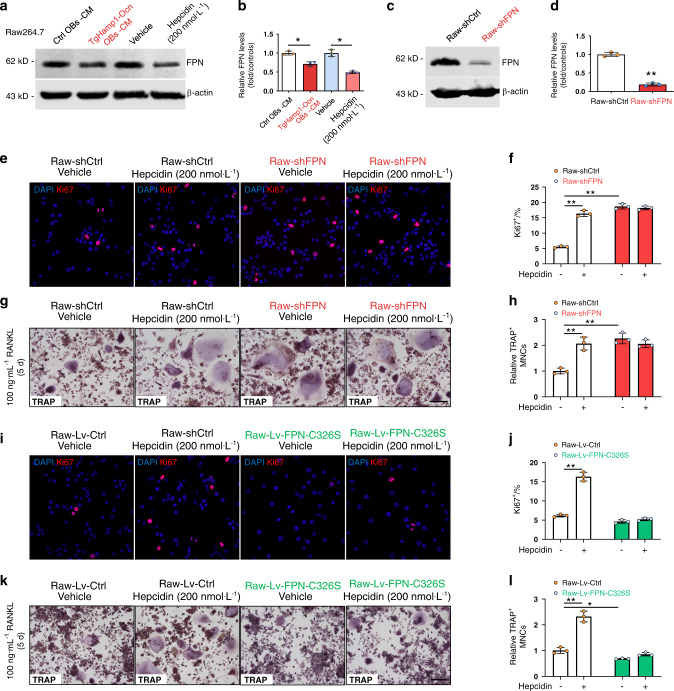
FPN regulates the proliferation and differentiation of RAW264.7 cells. **a**, **b** FPN protein levels in RAW264.7 cells after 4 h of treatment with Ctrl OB-CM, TgHamp1-Ocn OB-CM, vehicle or 200 nmol·L^−1^ hepcidin peptide. FPN levels were analyzed by western blotting. Representative blots are shown in (**a**). Quantitative data (the mean ± SD of three separate experiments) are presented in (**b**). *P < 0.05. **c**, **d** Western blot analysis of FPN expression in Ctrl and FPN-KD RAW264.7 cells. Quantitative data (the mean ± SD of three separate experiments) are presented in (**d**). **P < 0.01. **e**, **f** Immunostaining analyses of Ki67 in RAW264.7 cells. Ctrl and FPN-KD RAW264.7 cells were treated with vehicle or hepcidin (200 nmol·L^−1^) for 1 d. Quantitative data are shown in **f**. The data are presented as the mean ± SD of three separate experiments. **P < 0.01. **g**, **h** TRAP staining of RAW264.7 cells. Ctrl and FPN-KD RAW264.7 cells were treated with vehicle or hepcidin (200 nmol·L^−1^) and 100 ng·mL^−1^ RANKL for 5 d. Bar, 200 μm. Quantification of TRAP^+^ MNCs is shown in **h**. The data are presented as the mean ± SD of three separate experiments; **P < 0.01. **i**, **j** Immunostaining analyses of Ki67 in RAW264.7 cells. Lv-Ctrl and Lv-FPN-C326S-transfected cells were treated with vehicle or hepcidin (200 nmol·L^−1^) for 1 d. Quantitative data are shown in **j**. The data are presented as the mean ± SD of three separate experiments. **P < 0.01. **k**, **l** TRAP staining of RAW264.7 cells. Lv-Ctrl and Lv-FPN-C326S-transfected cells were treated with vehicle or hepcidin (200 nmol·L^−1^) and 100 ng·mL^−1^ RANKL for 5 d. Bar, 200 μm. The number of TRAP^+^ MNCs is shown in **l**. The data are presented as the mean ± SD of three separate experiments. *P < 0.05; **P < 0.01

It is known that hepcidin binds to FPN and promotes FPN internalization and degradation.^20^ Indeed, FPN levels, as determined by western blot analysis, were lower in RAW264.7 cells exposed to hepcidin^+^ CM or hepcidin peptide (200 nmol·L^−1^) for 4 h than in those treated with vehicle (Fig. 5a, b). We then asked whether suppressing FPN expression with shRNA in RAW264.7 cells can mimic the effect of hepcidin on cell proliferation and OC differentiation. A lentiviral plasmid encoding FPN shRNA (m) was generated, which sufficiently suppressed FPN expression (Fig. 5c, d). As expected, the Ki67^+^ proliferative cell density was increased in RAW-shFPN cells compared with RAW-shCtrl cells (Fig. 5e, f). Upon RANKL treatment (100 ng·mL^−1^, 5 d), more TRAP^+^ OCs were formed in cultured RAW-shFPN cells than in cultured Raw-shCtrl cells (Fig. 5g, h). These results thus demonstrate that FPN-KD has a similar effect as hepcidin in promoting macrophage proliferation and OC differentiation, implicating hepcidin-induced downregulation of FPN expression as a potential pathway for osteoclastogenesis. This finding was further supported by the failure of hepcidin to increase cell proliferation and differentiation in RAW-shFPN cells (Fig. 5e–h).

Notably, that a mutation in FPN (substitution of a serine residue for the cysteine residue at position 326) causes resistance to hepcidin binding and results in iron overload in vital organs.^47–50^ To further address the role of the “hepcidin-FPN regulatory axis” in osteoporosis, we generated a plasmid expressing the FPN-C326S point mutation. Compared with FPN-WT, FPN-C326S showed more stable expression after hepcidin treatment (Supplementary Fig. S11a, b). We then cloned this mutant protein into a lentiviral vector and generated RAW 264.7 cells stably expressing FPN-C326S-EGFP (Supplementary Fig. S11c–e). As shown in Fig. 5, the formation of TRAP^+^ cells was decreased in cells expressing the FPN-C326S mutation compared with control cells (Fig. 5k, l). In addition, hepcidin treatment promoted both the proliferation and differentiation of control RAW264.7 cells but had no effect on FPN-C326S-EGFP-expressing cells (Fig. 5i–l). These results further suggest that the hepcidin-FPN regulatory axis plays an important role in OC proliferation and differentiation.

### Iron accumulation in macrophages, an important mediator of the hepcidin-induced increase in macrophage proliferation

FPN, as the sole cellular iron exporter, controls intracellular iron levels, and downregulation of FPN expression may result in increased intracellular iron levels.^20^ We thus measured intracellular Fe^2+^ levels in RAW264.7 cells treated with or without hepcidin. As expected, intracellular Fe^2+^ levels were higher in RAW264.7 cells exposed to hepcidin^+^ CM or hepcidin peptide than in those treated with vehicle (Supplementary Fig. S12a). A similar increase in intracellular Fe^2+^ levels was also observed in RAW-shFPN cells (Supplementary Fig. S12b). These results are in line with the view that the hepcidin-FPN axis regulates intracellular iron homeostasis.

We then assessed whether increased intracellular iron levels underlie the hepcidin-induced increase in BMM or RAW264.7 cell proliferation. To this end, wild-type BMMs and RAW264.7 cells were treated with vehicle, 10 μmol·L^−1^ DFO (an iron chelator) or 100 μmol·L^−1^ FAC (an iron mimic) and EdU for 4 h and subjected to immunostaining analysis using antibodies. As shown in Supplementary Fig. S12c and d, the EdU^+^ BMM cell density was reduced by the iron chelator DFO (10 μmol·L^−1^) but increased by the iron mimic FAC (100 μmol·L^−1^). In line with these results, DFO decreased the EdU^+^ RAW264.7 cell density but FAC increased the density of these cells,(Supplementary Fig. S12e, f). These results support a role for intracellular iron in macrophage proliferation.

We next examined whether the increase in intracellular iron levels is necessary for hepcidin-mediated promotion of macrophage proliferation. Wild-type BMMs and RAW264.7 cells were treated with vehicle and 200 nmol·L^−1^ hepcidin peptide or 200 nmol·L^−1^ hepcidin peptide and 10 μmol·L^−1^ DFO (in the presence of EdU) for 4 h. Immunostaining analyses of EdU, Ki67 and pH3 showed increased densities of EdU^+^, Ki67^+^, and pH3^+^ macrophages upon hepcidin treatment (Supplementary Fig. S12g–l). In the presence of the iron chelator DFO, this hepcidin-induced increase in macrophage proliferation was abolished (Supplementary Fig. S12g–l), demonstrating the necessity of intracellular iron in this hepcidin-mediated effect.

### Increase in hepcidin expression in TgAPPswe-Ocn mice and APPswe^+^ OB lineage cells induced by ER stress

**Fig. 6:**
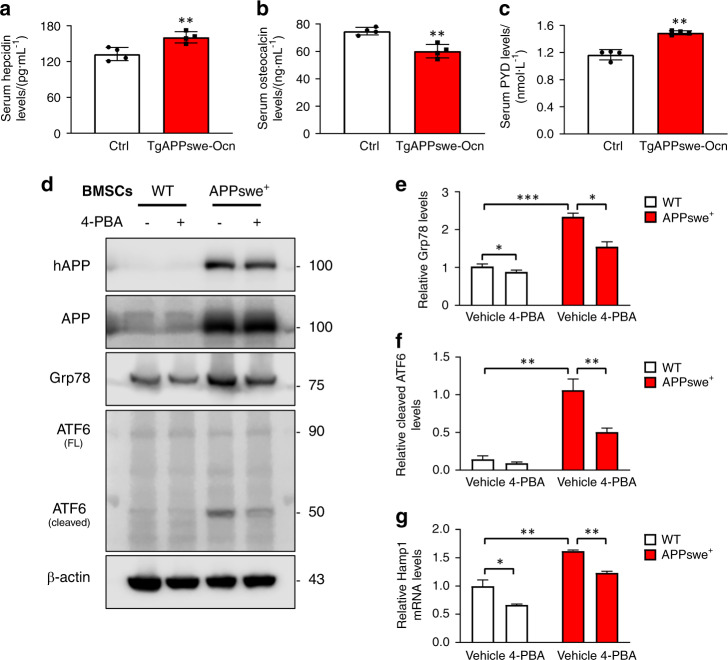
ER stress induces the expression of hepcidin in APPswe^+^ BMSCs. **a** Analysis of serum hepcidin levels in 3-month-old ctrl and TgAPPswe-Ocn mice by ELISA. **P < 0.01 (significant difference). **b** Analysis of serum osteocalcin (Ocn) levels in 3-month-old ctrl and TgAPPswe-Ocn mice by ELISA. The mean ± SD of measurements from four males per genotype are shown. **P < 0.01 (significant difference). **c** Elisa analysis of serum pyridinoline (PYD) levels. The values of mean ± SD (*n* = 4) were presented. **P < 0.01 (significant difference). **d** Western blot analysis of the expression of the indicated proteins in BMSCs from mice of the indicated genotypes (at the age of 3 months). Cells were treated with vehicle or 1 mmol·L^−1^ 4-PBA for 24 h. β-Actin was used as a loading control. **e**, **f** Quantitative analysis (mean ± SD; *n* = 3). *P < 0.05; **P < 0.01; ***P < 0.001. **g** Real-time PCR analysis of hepcidin expression in WT and APPswe^+^ BMSCs cultured with or without 4-PBA (1 mmol·L^−1^). *P < 0.05; **P < 0.01 (significant difference)

Like in Tg2576 mice, we investigated APPswe’s function in bone homeostasis in TgAPPswe-Ocn mice, which selectively express APPswe in OB lineage cells.^9,16^ TgAPPswe-Ocn mice show osteoporotic changes similar to those exhibited by Tg2576 mice.^16^ We thus measured serum levels of hepcidin in 3-month-old TgAPPswe-Ocn mice. As in Tg2576 mice, serum hepcidin levels were increased significantly in TgAPPswe-Ocn mice (Fig. 6a). We then asked whether the increase in serum hepcidin levels in TgAPPswe-Ocn mice is correlated with bone resorption. Serum levels of osteocalcin and PYD in TgAPPswe-Ocn mice were measured. As shown in Fig. 6b, c, TgAPPswe-Ocn mice exhibited a decrease in bone formation and an increase in bone resorption. We further addressed how APPswe expression in OB lineage cells increases hepcidin expression. Notably, APPswe and Aβ can induce ER stress in neurons,^51,52^ and ER stress is one of the factors that has been found to induce hepcidin expression in the liver, spleen, and HepG2 cells.^32^ In light of these observations, we speculate that the increase in hepcidin expression may result from APPswe-induced ER stress. To confirm this hypothesis, primary WT BMSCs and APPswe^+^ BMSCs were cultured with or without sodium 4-phenylbutyrate (4-PBA, 1 mmol·L^−1^), an inhibitor of ER stress,^53^ for 24 h. Western blot analyses showed increased protein levels of Grp78 (a marker of ER stress) and cleaved ATF6 in APPswe^+^ BMSCs, suggesting that ER stress is enhanced in APPswe^+^ BMSCs (Fig. 6d–f). Upon 4-PBA treatment, Grp78 and cleaved ATF6 levels were reduced in APPswe^+^ BMSCs (Fig. 6d–f), demonstrating that 4-PBA has an inhibitory effect on ER stress. Remarkably, in both WT and APPswe^+^ BMSCs, hepcidin mRNA levels were decreased by 4-PBA treatment compared with those vehicle treatment (Fig. 6g), supporting this speculation.

### Requirement of hepcidin expression in APPswe^+^ OB lineage cells for induction of osteoclastogenesis

**Fig. 7:**
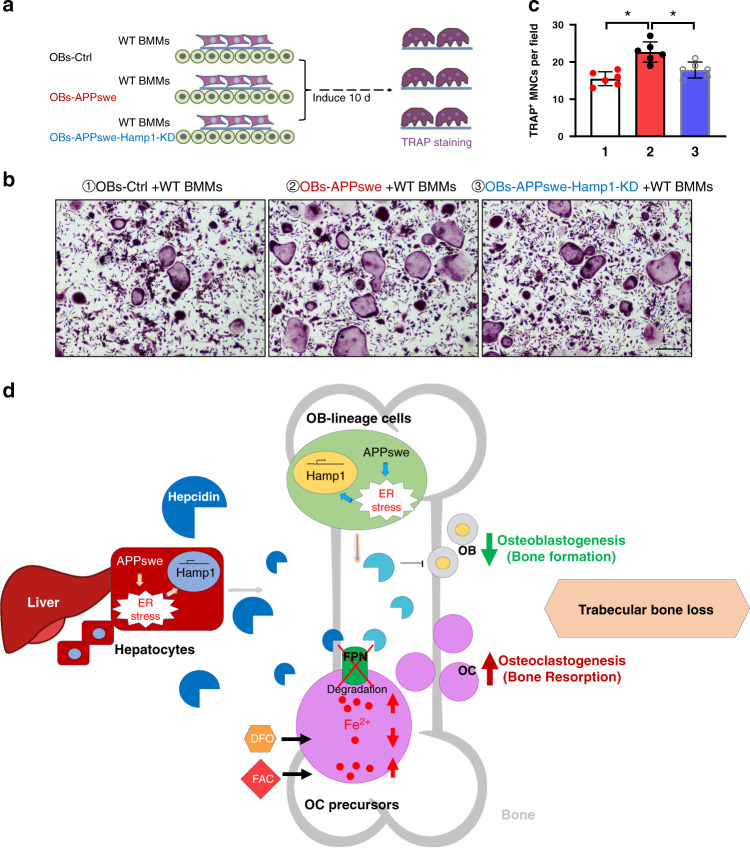
Hepcidin partially rescues the increase in osteoclastogenesis in the OB-APPswe-BMM coculture system. **a** Experimental strategy. **b** TRAP staining of OCs cocultured with OBs. Bar, 200 μm. **c** Quantification of TRAP^+^ MNCs per field in (**b**). The data are presented as the mean ± SD of six different coculture experiments. *P < 0.05. **d** Illustration of the working model. Hepcidin expression in hepatocytes or OB lineage cells induced by APPswe-driven ER stress increases osteoclastogenesis, which appears to be due to its role in downregulating FPN expression-mediated iron export in macrophages and thus increasing intracellular iron levels, proliferation of OC precursors, and OC differentiation

Given that APPswe increases hepcidin expression in OB lineage cells (Fig. 6g) and hepcidin promotes osteoclastogenesis (Fig. 4), we asked whether APPswe-induced hepcidin expression in OB lineage cells contributes to the APPswe-mediated increase in osteoclastogenesis. To answer this question, a lentivirus encoding shRNA-hamp1 was generated (Supplementary Fig. S13a), which efficiently suppressed hepcidin expression in OB lineage cells (Supplementary Fig. S13b). WT BMMs were cocultured with WT BMSCs or Hamp1-KD-BMSCs, as illustrated in Fig. 7a. After 10 d of cocultures, cells on coverslips were subjected to TRAP staining. As shown in Fig. 7b, c, TRAP^+^ MNCs were more abundant in APPswe^+^ BMSC-BMM cocultures than in WT BMSC-BMM cocultures; however, this increase in osteoclastogenesis was diminished when BMMs were cocultured with Hamp1 KD BMSCs (Fig. 7b, c). These results suggest that hepcidin expression in OB lineage cells is required for APPswe-induced osteoclastogenesis.

## Discussion

In this study, we provide evidence for hepcidin as a critical regulator of osteoclastogenesis underlying OC-mediated bone resorption and trabecular bone loss in Tg2576 mice. Hepcidin expression is increased not only in the sera but also in the livers, muscles, and OB lineage cells of Tg2576 mice. Increased hepcidin expression in hepatocytes or OB lineage cells plays a similar role in enhancing osteoclastogenesis, bone resorption, and trabecular bone loss. Further mechanical studies led us to propose a working hypothesis, which is depicted in Fig. 7d. Hepcidin expression in hepatocytes or OB lineage cells induced by APPswe-driven ER stress increases osteoclastogenesis, which appears to be due to the role of hepcidin in decreasing FPN-mediated iron export in macrophages and thus increasing intracellular iron levels, proliferation of OC precursors, and OC differentiation.

AD is one of the most serious neurodegenerative disorders, and patients with AD commonly have increased bone fracture rates and reduced bone density.^1,2^ APPswe has been found to suppress OB differentiation and promote OC differentiation in mouse models, such as the Tg2576 mouse model; however, the mechanisms underlying the association between APPswe and these processes remain poorly understood.^9,15,16^ Previously, we demonstrated that Aβ-RAGE signaling in OC precursor cells appears to be one of the critical pathways underlying age-dependent OC formation and activation in Tg2576 mice.^15^ Here, we provide several lines of evidence that hepcidin in OB lineage cells not only inhibits OB-mediated bone formation but also promotes OC-mediated bone resorption, revealing another pathway underlying APPswe-induced bone loss. First, hepcidin expression is increased in the sera of Tg2576 mice and TgAPPswe-Ocn mice (Figs. 1c–g and 6a), and both mouse lines express APPswe in OB lineage cells.^15,16^ Second, hepcidin expression is induced not only in hepatocytes^23–25^ but also in OB lineage cells in Tg2576 mice (Supplementary Fig. S1). Third, hepcidin expression in APPswe^+^ OB lineage cells is required for the induction of OC differentiation (Fig. 7). Fourth, the expression of hepcidin in either hepatocytes or OB lineage cells is sufficient to induce osteoclastogenesis and trabecular bone loss (Figs. 2–4).

Tg2576 mice show bone formation deficits at 1 month of age^16,54^; however, TgHamp1-Ocn mice show an osteoporosis phenotype at the age of 3 months and older but not at the age of 1 month (Supplementary Fig. S5). This is likely due to age-dependent hepcidin expression in OB lineage cells in TgHamp1-Ocn mice. Hepcidin expression in this mouse line is largely controlled by Ocn-Cre, and Cre activity in Ocn-Cre mice is low in developing osteoblasts at young ages but high in mature osteoblasts in adulthood.^17,55^ Our data also showed an age-dependent increase in hepcidin expression in TgHamp1-Ocn mice (Supplementary Fig. S3). In contrast, Tg2576 mice express APPswe under the control of the hamster prion promoter, which is ubiquitously expressed in different tissue/cell types starting in the embryonic stage. Bone phenotypes appear to be dependent on transgene expression; thus, TgHamp1-Ocn mice exhibit different phenotypic onsets.

There is an imbalance in iron homeostasis in AD. Abnormal iron metabolism leads to the generation of hydroxyl radicals through the Fenton reaction, triggers oxidative stress reactions, causes damage to cell lipid, protein, and DNA structure and function, and ultimately leads to cell death.^39,56^ Excessive iron contributes to the deposition of β-amyloid and the formation of neurofibrillary tangles, which in turn promote the development of AD.^56,57^ Interestingly, hepcidin, the systemic iron-regulatory hormone, is increased in serum samples from AD animal models (Figs. 1 and 6a; Supplementary Fig. S1) as well as AD patients.^58^ However, how hepcidin expression is induced in AD remains elusive. Here, we provide evidence that APPswe-induced ER stress results in an increase in hepcidin expression (Fig. 6). This finding is in agreement with previous reports showing that ER stress and the associated unfolded protein response (UPR) are induced in neurons but not in glial cells in the AD brain;^51,52^ ER stress can induce an inflammatory response via different UPR transducers;^59^ and the accumulation of pathogenic misfolded proteins and the disruption of intracellular Ca^2+^ signaling are fundamental mechanisms that underlie the induction of ER stress, leading to neuronal cell death.^60^ This finding is also supported by reports showing that chemically induced ER stress can induce hepcidin gene expression in HepG2 cells.^32,61^ The regulation of hepcidin by ER stress may be an important mechanism linking osteoporosis and AD.

Increased hepcidin expression in the serum may cause decreased systemic iron levels by leading to inhibition of iron absorption from the intestine. We detected decreases in serum Fe^2+^ and Fe^3+^ levels in both TgHamp1-Alb and TgHamp1-Ocn mice compared with age-matched control mice (Supplementary Fig. S3). However, in aged WT mice, both serum hepcidin and iron levels are higher than those in young adult mice (Supplementary Fig. S2a–c). This age-dependent increase in endogenous hepcidin expression may be a feedback response to increased iron overload and inflammation in aged mice.

In addition to APPswe-induced bone loss, hepcidin may be involved in the development of osteoporosis associated with other risk factors, such as age and chronic inflammation. Interestingly, hepcidin levels are elevated in aged WT mice (Supplementary Fig. S2a). This age-dependent increase in serum hepcidin levels is associated with an elevation in serum levels of iron (Fe^2+^ and Fe^3+^) (Supplementary Fig. S2b, c). Thus, it is possible that this age-dependent increase in endogenous hepcidin expression is a feedback response to increased iron overload in aged WT mice. In addition to aging, hepcidin expression is induced by chronic inflammation,^62^ which is often elevated in patients with chronic inflammation, such as anemia of inflammation, nonalcoholic fatty liver disease, and inflammatory bowel disease.^63–65^ Interestingly, these hepcidin inducers (aging and chronic inflammation) are risk factors for both AD and osteoporosis. While we believe that hepcidin plays a role in APPswe-induced trabecular bone loss, this view does not exclude the possible contributions of other up/downregulated genes to APPswe-induced osteoporotic changes. Many cytokines play an important role in bone remodeling,^66^ such as IL-4 and IL-16, which exhibit increased serum levels in Tg2576 mice (Fig. 1c–e) and have been reported to promote osteoclast differentiation and activation.^67,68^ We hope to investigate the function of other up/downregulated genes in future studies.

While the function of hepcidin in iron homeostasis is well established, its role in bone homeostasis remains controversial. Hepcidin has been found to facilitate OC differentiation in bone marrow macrophage (BMM) cultures,^34^ suggesting that it plays a positive role in bone resorption and a negative role in bone mass homeostasis. In contrast, hamp1 gene knockout (KO) mice develop a low bone mass phenotype,^69^ suggesting that hepcidin plays a positive role in bone mass homeostasis. Thus, the exact functions of hepcidin in bone formation, resorption, and bone mass homeostasis remain to be investigated. Our observations of reduced trabecular bone mass in both TgHamp1-Alb and TgHamp1-Ocn mice at the age of 3 months (Fig. 2) support the view that hepcidin plays a negative role in trabecular bone homeostasis. Notably, this finding is different from that reported in a study by Shen et al.,^69^ in which hamp1 KO mice at the age of 7 months displayed a low bone mass phenotype. The difference may have resulted from differences in the ages of the mice examined. It is possible that in young adulthood, hepcidin plays a negative role in bone homeostasis, but at older age, it may have a positive role. It is also possible that hepcidin’s function is dosage dependent. At the physiological level, hepcidin promotes bone homeostasis, but under pathological conditions, a high level of hepcidin expression is induced, which has a negative effect. Thus, either loss or gain of hepcidin may affect bone homeostasis. It is necessary to analyze the bone phenotypes of hamp1-KO mice at a young adult age and in TgHamp1-Alb and TgHamp1-Ocn mice at an older age. We hope to carry out these experiments in future studies.

Recent studies have shown that FPN deletion in osteoclast precursors increases osteoclastogenesis and decreases bone mass in vivo and that phenotypes are more pronounced in female mice than in male mice.^36^ This study supports the important role of the “hepcidin-FPN-iron axis” in osteoclastogenesis. Another study reported that FPN^C326S^ mice, which express a mutant version of FPN that cannot be targeted for degradation, show reduced bone mass in the lumbar vertebra but not the femur.^49^ An in vitro experiment also showed reduced OC formation in BMMs from Fpn^C326S^ mice, which supports a role for FPN^C326S^ in suppressing OC differentiation and OB mineralization,^49^ which is in line with our results. However, the researchers also showed that Alizarin Red staining was reduced in OBs from FPN^C326S^ mice, which appears to be inconsistent with our finding (Supplementary Fig. S7). It is possible that too high (Fpn^C326S^) or too low (TgHamp1-Ocn mice) FPN iron exporter activity in OBs may be detrimental to OB function. In addition, the reduction in bone mass of the lumbar vertebra in Fpn^C326S^ mice suggests that we should address whether there is any change in the lumbar vertebra in TgHamp1^OCN^ mice, which will be examined in our future studies.

The reduction in trabecular bone mass in both TgHamp1-Alb and TgHamp1-Ocn mice appears to be due in large to increases in OC-mediated bone resorption. Our finding that hepcidin increases OC differentiation in vitro (Fig. 4) is in line with previous literature.^34^ This view is further supported by the observation that hepcidin increases RANKL signaling (Fig. 4i–n). In addition to increasing OC differentiation, we observed that hepcidin markedly elevated the proliferation of OC precursors (both BMMs and RAW264.7 cells) (Supplementary Fig. S10). We thus propose that hepcidin increases both the proliferation and differentiation of OC precursors, which may underlie its role in promoting OC formation in vitro and in vivo. Note that the hepcidin-induced increase in OC precursor proliferation is dose dependent (Supplementary Fig. S10). This effect of hepcidin is largely due to its role of downregulating FPN expression in BMMs or OC precursors (Fig. 5), as suppressing FPN expression in RAW264.7 cells results in a similar increase in cell proliferation as hepcidin and diminishes the effect of hepcidin (Fig. 5). In line with this view, the iron chelator inhibitor DFO, but not the iron mimic FAC promotes the proliferation of BMMs and RAW264.7 cells (Supplementary Fig. S12). Mounting evidence shows that excess iron facilitates osteoclastogenesis and increases the bone-resorbing activity of mature osteoclasts.^38^ Iron has been reported to induce oxidative stress and stimulate osteoclast differentiation via NF-κB signaling.^70^ Iron not only affects osteoclastogenesis but also influences mature osteoclast activity; for example, inhibition of TRAP activity in osteoclasts abolishes bone resorption, and TRAP is an iron-containing enzyme, the activity of which is dependent on ferric iron.^71^ Together, these results support the working hypothesis presented in Fig. 7d.

In summary, our results suggest a negative role for hepcidin in regulating bone homeostasis by promoting OC precursor proliferation and differentiation, implicate hepcidin in osteoblastic APPswe-induced osteoclastogenesis, and reveal a new mechanism underlying AD-associated osteoporosis.

## Materials and methods

### Ethics statement

All experimental procedures were approved by the Institutional Animal Care and Use Committee (IACUC) at Case Western Reserve University (2017-0121 and 2017-0115) and were performed according to US National Institutes of Health guidelines.

### Animals and reagents

Tg2576 mice, which express human APP695 with the KM670/671NL mutations (APPswe) under the control of a hamster prion promoter, were purchased from Taconic (Hudson, NY, USA).^37^ TgAPPswe mice were generated by the use of the pCCALL2 plasmid as described previously.^16^ In brief, the transcription of hAPPswe in TgAPPswe mice is controlled by the cytomegalovirus (CMV) promoter, but its translation is blocked by a loxP-STOP-loxP sequence.^16^ Thus, the expression of hAPPswe is Cre-dependent. Tg-Hamp1 transgenic mice were also generated by the use of the pCCALL2 plasmid. The Hamp1-myc sequence was cloned downstream of the loxp-STOP-loxp sites in the pCCALL2 plasmid. The construct was linearized using ScaI/SfiI and electroporated into ES cells. Positive ES cell clones were injected into C57BL6/J blastocysts. Successful expression of Hamp-1-myc was verified by PCR and western blotting. Albumin (Alb)-Cre mice were purchased from the Jackson Laboratory (stock no 003574). Ocn-Cre mice were kindly provided by Tom Clemens (Johns Hopkins Medical School). All mouse lines were backcrossed onto the C57BL/6 background, housed in a room with a 12-h light/dark cycle and provided ad libitum access to water and rodent chow diet (Harlan Tekled S-2335). Control littermates were used in parallel for each experiment. C57BL/6 mice of various ages were obtained from the NIA (NIH).

Rabbit polyclonal antibodies, including FPN (PA5-77470, Invitrogen), Ki67 (ab16667, Abcam), Grp78 (ab21685, Abcam), APP (2452 S, Cell Signaling), pErk1/2 (#4370 S, Cell Signaling), Erk1/2 (#9102 S, Cell Signaling), Runx2 (ab192256, Abcam) and phospho-IκBα (Cell Signaling Technology) antibodies, and mouse monoclonal antibodies, including p-Histone H3 (C-2) (sc-374669, Santa Cruz Biotechnology), β-actin (A5441, Sigma-Aldrich), ATF6 (NBP1-40256, Novus Biologicals), NFATC1 (MA3024, Thermo Fisher), IκBα (Cell Signaling Technology) and hAPP (6E10) antibodies, were used. Recombinant M-CSF and recombinant RANKL were gifts from Dr. X. Feng (University of Alabama at Birmingham, Birmingham, AL). Hepcidin peptide was purchased from Bachem (4062144). Other chemicals and reagents used in this study were of analytical grade.

### L-Series label-based antibody arrays

Blood samples were collected, allowed to clot for 30 min and centrifuged for 10 min at 3 000 r·min^−1^. The serum samples were frozen at −80 °C until use. Antibody arrays were performed using an L-Series Glass Slide Antibody Arrays Kit (AAM-SERV-LG, Raybiotech, USA) according to the manufacturer’s instructions. In brief, the serum was dialyzed before the biotin-labeling step. The primary amine groups of the proteins in the sample were biotinylated, and then dialysis was performed to remove free biotin. The newly biotinylated sample was added onto a glass slide and incubated at room temperature. After incubation with Fluorescent Dye-Streptavidin, the signals were visualized by fluorescence.

### Measurement of hepcidin levels

Hepcidin levels were measured with a mouse hepcidin ELISA kit (LS-F11620; LifeSpan BioScience, Inc.). In brief, to prepare cell or tissue lysates, cells or tissues were washed with PBS, resuspended in PBS, lysed by ultrasonication, and centrifuged at 1 500 × *g* for 10 min at 4 °C to remove cellular debris. The supernatants were collected for analysis. The total protein levels in the supernatants were measured with a PierceTM BCA Protein Assay Kit (23225; Thermo Scientific). For blood/plasma samples, blood samples were collected, allowed to clot for 30 min, and centrifuged for 10 min at 3 000 r·min^−1^. Serum/plasma samples were frozen at −80 °C until use. The hepcidin levels were measured according to the manufacturer’s instructions. The hepcidin concentrations were calculated by comparing the readings against standard curves.

### Measurements of serum levels of osteocalcin and PYD

Osteocalcin and PYD levels were measured with a MicroVue Serum Osteocalcin Enzyme Immunoassay (EIA) kit (8002; Quidel Corporation) and a MicroVue Serum PYD EIA Kit (8019; Quidel Corporation), respectively, as described previously.^16,43^ The concentrations of osteocalcin and PYD were determined by comparing the readings against standard curves.

### μCT analysis

Excised femurs from mice were scanned using the Scanco µCT40 desktop cone-beam micro-CT scanner (Scanco Medical AG, Brüttisellen, Switzerland) using µCT Tomography (v5.44). The scans were automatically reconstructed into 2-D slices, and all slices were analyzed using the µCT Evaluation Program (v.6.5-2, Scanco Medical).

Each femur was placed inverted in a 12-mm diameter scanning holder and scanned at the following settings: 12 µm resolution, 55 kVp, 145 µA, and an integration time of 200 ms. For cortical analysis, the bone was scanned at the midshaft to generate 25 slices. The region of interest (ROI) was drawn on every slice and fitted to the outside of the cortical bone to include all the bone and marrow. The threshold for cortical bone was set at 621 mgHA·ccm^−1^. 3-D reconstruction (µCT Ray (v3.8)) was performed using all the outlined slices. Bone volume (BV), total volume (TV), BV/TV, bone density, and cortical thickness data were obtained.

For trabecular bone, the scan was started at the growth plate and consisted of 211 slices. The region of interest was outlined where the condyles ended, and 100 slices were outlined from this point on the inside of the cortical bone, encompassing only the trabecular bone and marrow. Trabecular bone was thresholded at 414 mgHA·ccm^−1^, and 3-D analysis was performed on 100 slices. Bone volume, density, total volume, trabecular number, thickness and separation data were obtained.

An additional scan was performed to image the whole femur. Femurs were placed horizontally in a 20-mm scanning holder and scanned at the following settings: 20 µm resolution, 55 kVp, 145 µA, and an integration time of 200 ms. Approximately 210 slices were taken. The region of interest (whole bone) was outlined in all slices that contained bone. The outlines included all parts of the femur. A threshold of 414 mgHA·ccm^−1^ was used to distinguish bone, and bone volume and density data were obtained.

### Bone histomorphometric analysis

Bone histomorphometric analyses were performed as previously described.^16,43^ In brief, mouse tibias and femurs were fixed overnight in 10% formalin, decalcified in 14% EDTA, embedded in paraffin, sectioned, and subjected to hematoxylin and eosin and TRAP staining (Acid Phosphatase, Leukocyte (TRAP) Kit; 387A-1KT; Sigma-Aldrich). Morphometric perimeters were determined by measuring areas situated at least 0.5 mm from the growth plate excluding the primary spongiosa and trabeculae connected to the cortical bone.

#### Dynamic bone histomorphometry to measure the rate of bone formation in vivo

Briefly, mice (P80) were injected (intraperitoneally) with fluorochrome-labeled calcein green (10 mg·kg^−1^, Sigma-Aldrich) twice (10-d interval). The mice were sacrificed 2 d after the second injection. The left tibias and femurs were fixed in 70% (vol/vol) ethanol overnight, embedded in methyl methacrylate, and sectioned at 7–10 μm thickness. Images were obtained using a Zeiss LSM 800 fluorescence microscope. The mineral apposition rate (MAR) in μm·d^−1^ and bone formation rate (BFR) [BFR = MAR × MS (mineral surface) / BS (bone surface)] were calculated by measuring double fluorescence at the trabecular bone and endocortical surfaces.

### In vitro osteoblast (OB)/osteoclast (OC) lineage cell cultures

Whole bone marrow cells were flushed from the long bones of Ctrl, TgHamp1-Ocn, and TgHamp1-Alb mice and plated in 100-mm culture plates in DMEM supplemented with 10% fetal bovine serum (FBS) and 1% penicillin/streptomycin for 3 d.

For OB lineage cultures, the culture medium in plates with adherent cells was replaced with fresh culture medium every 3 d. After 7 d of passaging by trypsin digestion, 1 × 10^5^ per cm^2^ bone marrow stromal cells (BMSCs) were plated on coverslips containing DMEM supplemented with 10% FBS, 1% penicillin/streptomycin, 10 mmol·L^−1^ β-glycerophosphate disodium salt hydrate (G9422, Sigma-Aldrich) and 50 μmol·L^−1^ L-ascorbic acid-2-phosphate sesquimagnesium salt hydrate (A8960, Sigma-Aldrich), the cultured medium was replaced with fresh culture medium every 3 d. The cells were then subjected to ALP staining (Leukocyte Alkaline Phosphatase Kit; 85L3R-1KT; Sigma-Aldrich) to confirm their OB identity on day 7 and day 14. After 21 d, to visualize the calcified matrix, Alizarin Red S staining and quantification were performed.

For OC lineage cultures, nonadherent cells were harvested and subjected to Ficoll–Hypaque gradient centrifugation for purification of bone marrow macrophages (BMMs). The cells were plated in 100-mm culture dishes in α-MEM supplemented with 10% FBS, 1% penicillin/streptomycin, and 10 ng·mL^−1^ recombinant M-CSF.

For osteoclastogenesis, 1 × 10^5^ per cm^2^ BMMs were incubated with OC differentiation medium containing 10 ng·mL^−1^ recombinant M-CSF and 100 ng·mL^−1^ recombinant RANKL. Mature OCs began to form on days 4 to 5 after RANKL treatment. The cells were then subjected to TRAP staining (Acid Phosphatase, Leukocyte (TRAP) Kit; 387A-1KT; Sigma-Aldrich) to confirm their OC identity on day 7.

### Bone resorption activity assay

BMMs or RAW264.7 cells were plated in 24-well plates (COSMO BIO USA) coated with calcium phosphate and incubated with OC differentiation medium supplemented with 10 ng·mL^−1^ M-CSF and 100 ng·mL^−1^ RANKL for 8 d. The culture medium was changed every other day. On day 8, the plate was washed with bleach (5% sodium hypochlorite) for 5 min to remove the cells, washed three times with distilled water, and dried. Putative resorption pits were visualized with a microscope. The data were analyzed using ImageJ software.

### In vitro primary OB cultures

Primary OB cultures were prepared from the long bones of 3-month-old Ctrl, TgHamp1-Ocn or Tg2576 mice. In brief, small bone pieces were incubated in collagenase solution to remove all remaining soft tissue and adherent cells and then transferred to 60-mm culture dishes containing DMEM supplemented with 10% FBS, 1% penicillin/streptomycin, 10 mmol·L^−1^ β-glycerophosphate disodium salt hydrate and 50 μmol·L^−1^ L-ascorbic acid-2-phosphate sesquimagnesium salt hydrate. The culture medium was replaced three times per week. Bone cells started to migrate from the bone chips after 3–5 d. After 2 weeks, the monolayer was trypsinized by incubating the cells with trypsin solution.

For conditioned medium (CM) treatment, OBs were plated in 100-mm tissue culture plates in DMEM supplemented with 10% FBS, 1% penicillin/streptomycin, 10 mmol·L^−1^ β-glycerophosphate disodium salt hydrate and 50 μmol·L^−1^ L-ascorbic acid-2-phosphate sesquimagnesium salt hydrate (A8960, Sigma-Aldrich). BMMs derived from WT mice were placed on presterilized glass coverslips in 12-well plates. CM from plates containing OBs, 10 ng·mL^−1^ recombinant M-CSF and 100 ng·mL^−1^ recombinant RANKL were added to 12-well plates containing BMMs every other day. After 7 d, the cells were subjected to TRAP staining or immunofluorescence staining.

### Cell lines and lentiviruses

RAW264.7 cells were maintained in DMEM supplemented with 10% FBS and 1% penicillin/streptomycin. To induce osteoclastogenesis of RAW264.7 cells, 1 × 10^5^ per cm^2^ cells were incubated with α-MEM supplemented with 100 ng·mL^−1^ recombinant RANKL. Mature OCs began to form on days 2 to 3 after RANKL treatment. The cells were then subjected to TRAP staining on day 5.

Ferroportin-1 (FPN) shRNA (m) (shRNA-FPN) lentiviral particles were purchased from Santa Cruz Biotechnology, Inc (sc-60634-V). Raw-shCtrl and Raw-shFPN cell lines were obtained by infecting RAW264.7 cells with lentiviral particles encoding scramble control shRNA and shRNA-FPN, respectively. In brief, the cells were infected with lentiviral particles for 1 d in 2 μg·mL^−1^ polybrene medium. On day 3, the culture medium was removed and replaced with complete medium (without polybrene). After 5–6 d, stable clones expressing shRNA were selected with 5 μg·mL^−1^ puromycin dihydrochloride, which induced the death of untransduced cells.

To generate Hamp1 shRNA lentiviral particles, sense (GCAGAACAGAAGGCATGATGG) and antisense Hamp1-shRNA were synthesized, annealed, and then cloned into the pLL3.7 lentiviral vector, which expresses shRNA under the mouse U6 promoter. A CMV-EGFP reporter cassette was included in the vector to monitor expression. The lentivirus was packaged in HEK293 cells and purified. Cultured OBs derived from Tg2576 mice were infected with Hamp1-shRNA or scramble shRNA lentiviral particles and purified by fluorescence-activated cell sorting (FACS).

To generate FPN-C326S-EGFP lentiviral particles, we first generated a FPN-C326S point mutation (TGC-AGC) in the PLX304-SLC40A1 construct (DNASU, HsCD00442615) by using the Q5 Site-Directed Mutagenesis Kit (E0554, New England Biolabs, Inc.). Primers with the following sequences were used to generate the point mutation: GGGCTCTGACaGCATCACCAC and AGGACAGTCATATAAAGGAAAGC. FPN-C326S was then subcloned into the pLv-hPGK-EGFP lentiviral vector. The lentivirus was packaged in HEK293 cells and purified. RAW264.7 cells were infected with lentiviral particles and purified by fluorescence-activated cell sorting (FACS).

### OB-BMM coculture assays

For OB/BMM coculture, primary OB cultures were prepared from the long bones of 3-month-old Ctrl and Tg2576 mice. The OBs were infected with Hamp1-shRNA or scramble shRNA lentiviral particles and purified by FACS. Purified WT OBs, APPswe OBs, and APPswe-Hamp1 D OBs were cultured in 100-mm culture dishes containing α-MEM supplemented with 10% FBS, 1% penicillin/streptomycin, 10 mmol·L^−1^ β-glycerophosphate disodium salt hydrate and 50 μmol·L^−1^ L-ascorbic acid-2-phosphate sesquimagnesium salt hydrate. BMMs derived from WT mice were placed on presterilized glass coverslips in 12-well plates. The coverslips were then transferred to 100-mm plates containing OBs. The culture medium was replaced three times per week. After 10 d, the cells on the coverslips were subjected to TRAP staining.

### Cell lysis and western blot analysis

Cells were lysed in lysis buffer containing 50 mmol·L^−1^ Tris-HCl (pH 7.5) 150 mmol·L^−1^ NaCl, 1% (vol/vol) Triton X-100, 0.1% SDS, 0.5% deoxycholate, and 1 mmol·L^−1^ EDTA supplemented with protease inhibitors (1 μg·mL^−1^ leupeptin and pepstatin, 2 μg·mL^−1^ aprotinin, and 1 mmol·L^−1^ PMSF) and phosphatase inhibitors (10 mmol·L^−1^ NaF and 1 mmol·L^−1^ Na_3_VO_4_). Whole-cell extracts were fractionated by SDS-PAGE and transferred to a nitrocellulose membrane (1620112, Bio-Rad Laboratories). After incubation with 5% BSA in TBST (10 mmol·L^−1^ Tris, 150 mmol·L^−1^ NaCl, and 0.5% Tween 20; pH 8.0) for 1 h, the membrane was incubated with the indicated antibodies overnight at 4 °C. The membrane was washed with TBST three times and incubated with a 1:2 000 dilution of horseradish peroxidase-conjugated anti-mouse or anti-rabbit antibodies for 1 h. The blot was washed with TBST three times and visualized with the Li-Cor system.

### RNA isolation and qPCR

Total RNA was isolated by TRIzol extraction (15596018; Invitrogen). Quantitative PCR (qPCR) was performed using a QuantiFast SYBR Green PCR Kit (204057; QIAGEN) on a qPCR system (StepOne Plus). The following primers were used: GAPDH, 5′-AGGTCGGTGTGAACGGATTTG-3′ and 5′ TGTAGACCATGTAGTTGAGGTCA-3′; Hamp1, 5′-TGT CTGCCCTGCTTTCTT-3′ and 5′-CTGCCTGTCTCCTGCTTC-3′; NFATC1, 5′-GTCATCGGCGGGAAGAAG-3′ and 5′-TGGTTGCGGAAAGGTGGT-3′; Runx2, 5′-AACTTCCTGTGCTCCGTG-3′ and 5′-CGTTGAACCTGGCTACTT-3′; SP7, 5′-GGAAAGGAGGCACAAAGA-3′ and 5′-AGGGAAGGGTGGGTAGTC-3′; Col1a1, 5′-GAGGGCGAGTGCTGTGCT-3′ and 5′-CCAGGCTGTCCAGGGATG-3′; and Opn, 5′-TTTCACTCCAATCGTCCC-3′ and 5′-GTGGCATCAGGATACTGTTCAT-3′.

### Immunofluorescence staining and imaging analysis

BMMs from WT mice and RAW264.7 cells were plated on coverslips at a density of 5 × 10^4^ per cm^2^, allowed to recover overnight and divided into 4 groups. Cells in group A were incubated with CM from control or TgHamp1-Ocn OBs for 3 h. Cells in group B were incubated with vehicle, 20 nmol·L^−1^ hepcidin peptide (4062144; Bachem), 200 nmol·L^−1^ hepcidin peptide or 2 000 nmol·L^−1^ hepcidin peptide for 3 h. Cells in group C were incubated with vehicle, 10 μmol·L^−1^ deferoxamine mesylate salt (DFO) (D9533; Sigma-Aldrich) or 100 μmol·L^−1^ ammonium iron(III) citrate (FAC) (F5879; Sigma-Aldrich) for 3 h. Cells in group D were incubated with vehicle, 200 nmol·L^−1^ hepcidin peptide or 200 nmol·L^−1^ hepcidin peptide with 10 μmol·L^−1^ DFO for 3 h. Ctrl and FPN-depleted RAW264.7 cells were plated on coverslips at a density of 5 × 10^4^ per cm^2^ and then allowed to recover overnight. Then, we used the iClick^TM^ EdU Andy Fluor 488 Imaging Kit (A003; GeneCopoeia^TM^). EdU solution (2X) was prewarmed and then added to medium containing the cells to be treated so that the final concentration was 1X. The cells were incubated for 1 h, and then cell fixation, permeabilization and EdU detection were performed according to the manufacturer’s instructions. Then, the coverslips were incubated with the indicated antibodies overnight at 4 °C. The coverslips were washed three times with PBS and incubated with a 1:500 dilution of anti-mouse or anti-rabbit antibodies and 0.5 μg·mL^−1^ DAPI for 1 h. The coverslips were washed with PBS three times, mounted with Vectashield mounting medium (H-1000; Vector Laboratories) and imaged with a Zeiss LSM 800 confocal microscope at room temperature. Fluorescence quantification was performed using Zen software according to the manufacturer’s instructions (Zeiss).

### Intracellular ferrous iron (Fe^2+^) and total iron (Fe^2+^ & Fe^3+^) level measurements

Intracellular iron levels were measured using an iron assay kit (MAK025-1KT; Sigma-Aldrich). In brief, the cells were rapidly homogenized in 4 volumes of iron assay buffer and centrifuged at 16 000 × *g* for 10 min at 4 °C to remove insoluble material. Ferrous iron (Fe^2+^) and total iron (Fe^2+^&Fe^3+^) levels were analyzed using a kit according to the manufacturer’s instructions.

### Statistical analysis

All data are expressed as the mean ± SD. The data were analyzed by Student’s *t*-test or two-way ANOVA followed by post hoc tests (GraphPad Software Prism 8). The significance level was set at *P* < 0.05 (*P < 0.05, **P < 0.01, and ***P < 0.001).

## Supplementary information

Supplementary Information

Supplementary Fig S1

Supplementary Fig S2

Supplementary Fig S3

Supplementary Fig S4

Supplementary Fig S5

Supplementary Fig S6

Supplementary Fig S7

Supplementary Fig S8

Supplementary Fig S9

Supplementary Fig S10

Supplementary Fig S11

Supplementary Fig S12

Supplementary Fig S13
