# Supplementary material for: Hepcidin contributes to Swedish mutant APP-induced osteoclastogenesis and trabecular bone loss
Source: Bone Res. 2021 Jun 9;9:31. doi: 10.1038/s41413-021-00146-0 (PMC8190093; doi:10.1038/s41413-021-00146-0)
Supplement: Supplementary file 1 — Supplementary Information [file 41413_2021_146_MOESM1_ESM.docx]

**Supplementary information**

**Supplementary Table S1. The list of the 90 proteins in the label-based antibody arrays.**

**Supplementary Figure S1. Up-regulation of Hepcidin expression in liver, muscle and BMSCs of 3-MO Tg2576 mice.**

**a** Real-time PCR analysis of Hepcidin expression in different tissue of 3-MO WT and Tg2576 mice. * P < 0.05, significant difference.

**b** Elisa analysis of Hepcidin expression. * P < 0.05, significant difference.

**Supplementary Figure S2. Up-regulating of Hepcidin expression in aging mice.**

**a** ELISA analyses of serum hepcidin of 3-MO, 6-MO, 9-MO, 12-MO, 18-MO, 24-MO and 30-MO old male C57BL/6J mice. The values of mean±SD from four different mice of each age and in relative to 3-MO mice group are shown. * P<0.05, ** P<0.01, *** P<0.001.

**b, c** Intracellular ferrous iron (Fe^2+^) and total iron (Fe^2+^ & Fe^3+^) analyses of 3-MO, 6-MO, 9-MO, 12-MO, 18-MO, 24-MO and 30-MO old male C57BL/6J mice. The values of mean±SD from four different mice of each age and in relative to 3-MO mice group are shown. ** P<0.01, *** P<0.001.

**d, e** Elisa analyses of serum osteocalcin and pyridinoline (PYD) of 3-MO, 6-MO, 9-MO, 12-MO, 18-MO, 24-MO and 30-MO old male C57BL/6J mice. The values of mean±SD from four different mice of each age and in relative to 3-MO mice group are shown. * P<0.05, ** P<0.01.

**Supplementary Figure S4. Generation of TgHamp1-Alb and TgHamp1-Ocn mice.**

**a** Illustration of breeding strategies of generation of TgHamp1-Alb and TgHamp1-Ocn mice.

**b** Photos of 3-MO TgHamp1-Alb and TgHamp1-Ocn mice. The littermates Cre or Tg-Hamp1 mice were used as the control.

**c-f** Body lengths and body weights of 3-MO TgHamp1-Alb and TgHamp1-Ocn littermates. Values are presented as mean±SD (n = 5). NS, no significant difference.

**g-j** ELISA analysis of hepcidin in liver lysate, osteoblasts (OBs), and serum from 3-MO male TgHamp1-Alb / TgHamp1-Ocn and Ctrl littermates. Mean±SD values from four different mice of each genotype and in relative to Ctrl group are shown. * P<0.05, ** P<0.01, *** P<0.001.

**k-n** Intracellular ferrous iron (Fe^2+^) and total iron (Fe^2+^ & Fe^3+^) analyses of 3-MO male TgHamp1-Alb / TgHamp1-Ocn and Ctrl littermates. The values of mean±SD from four different mice. * P<0.05, ** P<0.01.

**Supplementary Figure S4. Decreased trabecular bone mass in 3-MO, not 1-MO, TgHamp1-Alb mice.**

**a** H&E staining analysis of femur sections in 1-MO Ctrl and TgHamp1-Alb male mice. Bar, 300μm.

**b-e** Quantitative analyses are shown in (b-e) as mean±SD (n = 3 femur samples for each genotype). **, P<0.01; NS, no significant difference.

**f** H&E staining analysis of femur sections in 3-MO Ctrl and TgHamp1-Alb male mice. Bar, 300μm.

**g-j** Quantitative analyses are shown in (g-j) as mean±SD (n = 5 femur samples for each genotype). * P<0.05; ** P<0.01; NS, no significant difference.

**k** H&E staining analysis of femur sections in 3-MO Ctrl and TgHamp1-Alb female mice. Bar, 300μm.

**l-o** Quantitative analyses are shown in (l-o) as mean±SD (n = 4 femur samples for each genotype). ** P<0.01; NS, no significant difference.

**Supplementary Figure S5. Decreased trabecular bone mass in 3-MO, not 1-MO, TgHamp1-Ocn mice.**

**a** H&E staining analysis of femur sections in 1-MO Ctrl and TgHamp1-Ocn male mice. Bar, 300μm.

**b-e** Quantitative analyses are shown in B-E as mean±SD (n = 3 femur samples for each genotype). ** P<0.01; NS, no significant difference.

**f** H&E staining analysis of femur sections in 3-MO Ctrl and TgHamp1-Ocn male mice. Bar, 300μm.

**g-j** Quantitative analyses are shown in g-j as mean±SD (n = 5 femur samples for each genotype). * P<0.05; ** P<0.01; NS, no significant difference.

**k** H&E staining analysis of femur sections in 3-MO Ctrl and TgHamp1-Ocn female mice. Bar, 300μm.

**l-o** Quantitative analyses are shown in (l-o) as mean±SD (n = 4 femur samples for each genotype). * P<0.05, ** P<0.01; NS, no significant difference.

**Supplementary Figure S6. Decreased trabecular bone formation rate in 3-MO TgHamp1-Ocn, not TgHamp1-Alb, mice.**

**a** Representative images of histologic sections showing calcein labeling of Tb and endocortical bone (Ec) in femur mid diaphysis of 3-MO male Ctrl and TgHamp1-Alb littermates. Bar, 50 μm.

**b-d** Quantitative analyses of MAR (mineral apposition rate) (b), MS (mineral surface) / BS (bone surface) (c), BFR (bone formation rate) (d) of Tb, Cb.E and Cb.P are presented respectively. The values of mean±SD from four different mice of each genotype are shown. NS, no significant difference.

**e** ELISA analysis of serum osteocalcin of 3-MO Ctrl and TgHamp1-Alb littermates. Five different mice of each genotype were examined and the values of mean±SD in relative to Ctrl group are shown. NS, no significant difference.

**f** Representative images of histologic sections showing calcein labeling of Tb and Ec in femur mid diaphysis of 3-MO male Ctrl and TgHamp1-Ocn littermates. Bar, 50μm.

**g-i** Quantitative analyses of MAR (g), MS/ BS (h), BFR (i) of Tb and Ec are presented respectively. The values of mean±SD from four different mice of each genotype are shown. NS, no significant difference.

**j** ELISA analysis of serum osteocalcin of 3-MO Ctrl and TgHamp1-Ocn littermates. Six different mice of each genotype were examined and the values of mean±SD in relative to Ctrl group are shown.

**Supplementary Figure S7. Decreased osteoblastogenesis of 3-MO TgHamp1-Ocn, not TgHamp1-Alb, mice in vitro.**

**a, e** ALP staining and Alizarin Red S staining analysis of cultured OBs derived from BMSCs of different genotypes. Representative images at days 7, 14 and 21 are shown.

**b, c, f, g** Quantitative analysis is shown as mean±SD from five different cell culture experiments. ** P<0.01; NS, no significant difference.

**d, h** Real-time PCR analysis of Runx2, SP7, Col1a1 and Opn expression in BMSCs derived from the indicated mice. Data are shown as mean ± SD, n = 3; *, P < 0.05.

**Supplementary Figure S8. Decreased BMSCs proliferation of 3-MO TgHamp1-Ocn mice.**

**a, c** BMSCs were derived from 3-MO TgHamp1-Alb, TgHamp1-Ocn mice and their control mice. Representative images of immunostaining of Ki67 and cleaved caspase 3 are shown in (a) and (c). No positive signal of cleaved caspase 3 was detected.

**b, d** Quantitative data of KI67+ cells in (a) and (c). ** P<0.01; NS, no significant difference.

**Supplementary Figure S9. Decreased trabecular Runx2+ cell number in 3-MO TgHamp1-Ocn mice.**

**a, c** Immunostaining analysis of Runx2 and SOST in trabecular region and cortical region of 3-MO TgHamp1-Alb, TgHamp1-Ocn and ctrl mice.

**b, d** Quantitative data of Runx2+ and SOST+ cells in (a) and (c). * P<0.05.

**Supplementary Figure S10. Hepcidin promotes the proliferation of BMMs and Raw264.7 cells.**

**a** Immunostaining analysis of EdU, Ki67 and pH3 of RAW 264.7 cells in the presence of Ctrl OBs-CM or TgHamp1-Ocn OBs-CM for 4 hours. Representative images are shown in (a). Bar, 50μm.

**b-d** Quantitative data of EdU+, Ki67+ and pH3+ cells in g. Data represent mean±SD from four separate experiments. * P<0.05, ** P<0.01.

**e** RAW 264.7 cells were treated with sham, hepcidin peptide 20 nM, 200 nM or 2000 nM for 4 hours respectively. After treatment, representative images of immunostaining of EdU, Ki67 and pH3 are shown in e. Bar, 50μm.

**f-h** Quantitative data of EdU+, Ki67+ and pH3+ cells in e. Data represent mean±SD from four separate experiments. * P<0.05; ** P<0.01; *** P<0.001; NS, no significant difference.

**i** WT BMMs were treated with vehicle, hepcidin peptide 20 nM, 200 nM or 2000 nM for 4 hours respectively. After treatment, representative images of immunostaining of EdU are shown in (i). Bar, 50μm.

**j** Quantitative data of EdU+ cells in (i). Data represent mean±SD from four separate experiments. ** P<0.01; NS, no significant difference.

**Supplementary Figure S11. Generation of Raw-FPN-C326S cell line.**

**a, b** Western blot analysis of FPN-C326S in HEK293 cells. HEK293 cells were transfected with FPN-C326S or FPN-WT plasmids, then treated with vehicle or Hepcidin (200 nM) for 2 days. Quantitative data was shown in b. * P<0.05.

**c** Map of FPN-C326S lentiviral particles. FPN-C326S was cloned into the pLV-hPGK-EGFP lentivirus vector.

**d, e** Raw264.7 cells were infected with control or FPN-C326S-EGFP lentivirus particles. Immunostaining analysis and western blot analysis were performed to confirm the expression of FPN-C326S-EGFP.

**Supplementary Figure S12. Iron is important for proliferation of BMMs and Raw 264.7 cells.**

**a** Intracellular ferrous iron (Fe^2+^) analyses in Raw 264.7 cells after 4-hour treatment with Ctrl OBs-CM, TgHamp1-Ocn OBs-CM, vehicle or 200 nM hepcidin peptide. Data represent mean±SD from three separate experiments. ** P<0.01.

**b** Intracellular ferrous iron (Fe^2+^) analyses of Ctrl and FPN-KD Raw 264.7 cells. Data represent mean±SD from three separate experiments. ** P<0.01.

**c** WT BMMs were treated with vehicle, 10 μM DFO or 100 μM FAC for 4 hours respectively. After treatment, representative images of immunostaining of EdU are shown. Bar, 50μm.

**d** Quantitative data of EdU+ cells in (c). Data represent mean±SD from four separate experiments. ** P<0.01.

**e** RAW 264.7 cells were treated with vehicle, 10 μM DFO or 100 μM FAC for 4 hours respectively. After treatment, representative images of immunostaining of EdU are shown. Bar, 50 μm.

**f** Quantitative data of EdU+ cells in (e). Data represent mean±SD from four separate experiments. ** P<0.01, *** P<0.001.

**g** Wildtype BMMs were treated with vehicle, 200 nM hepcidin peptide or 200 nM hepcidin peptide with 10 μM DFO for 4 hours respectively. After treatment, representative images of immunostaining of EdU are shown. Bar, 50μm.

**h** Quantitative data of EdU+ cells in (g). Data represent mean±SD from four separate experiments. **, P<0.01; NS, no significant difference.

**i** RAW 264.7 cells were treated with vehicle, 200 nM hepcidin peptide or 200nM hepcidin peptide with 10 μM DFO for 4 hours respectively. After treatment, representative images of immunostaining of EdU, Ki67 and pH3 are shown. Bar, 50μm.

**j-l** Quantitative data of EdU+, Ki67+ and pH3+ cells in (i). Data represent mean±SD from four separate experiments; **, P<0.01; ***, P<0.01; NS, no significant difference.

**Supplementary Figure S13. Generation of Hamp1-KD OBs.**

**a** Generation of shHamp1 lentiviral particles.

**b** ELISA analysis of hepcidin in Ctrl-OBs or Hamp1-KD-OBs. OBs were infected with shRNA-Hamp1 or scramble lentiviral particles and purified by FACS. Data represent mean±SD in relative to WT-OBs group and are from three separate experiments. **, P<0.01.

**Supplementary Table S1**

| **Protein name** | **Tg2576/Ctrl Fold-change** | **P value** | **Protein name** | **Tg2576/Ctrl Fold-change** | **P value** |
| --- | --- | --- | --- | --- | --- |
| Acrp30 | 1.080966 | 0.735633 | IL-3 | 0.908428 | 0.284051 |
| Alkaline phosphatase | 0.528571 | 0.058344 | IL-31 | 0.760943 | 0.39849 |
| Alpha-synuclein | 3.799198 | 0.007944 | IL-4 | 1.186982 | 0.023535 |
| Annexin A5 | 1.55458 | 0.04928 | IL-5 | 0.806645 | 0.307196 |
| b FGF | 1.240126 | 0.563511 | IL-6 | 0.746658 | 0.237857 |
| CCL1 | 1.169415 | 0.346781 | IL-7 | 0.849294 | 0.066177 |
| CFI | 2.067055 | 0.02794 | IL-9 | 1.360673 | 0.150275 |
| CRG2 | 2.22 | 0.006356 | ITAC | 1.024155 | 0.953788 |
| CRP | 0.823215 | 0.194631 | KC | 1.776316 | 0.168246 |
| CXCL13(BLC) | 0.919242 | 0.568893 | LIF | 1.264779 | 0.349087 |
| CXCL16 | 1.253906 | 0.388111 | L-Selectin | 1.175131 | 0.248523 |
| Cyclophilin A | 1.178068 | 0.226096 | MCP-1 | 2.920455 | 0.127279 |
| Cystatin | 1.182517 | 0.128052 | M-CSF | 1.121411 | 0.712822 |
| DKK-1 | 0.976103 | 0.909841 | MDC | 1.041667 | 0.946254 |
| EG-VEGF | 1.057705 | 0.636691 | MIG | 0.93617 | 0.834701 |
| Eotaxin | 1.072037 | 0.487806 | MIP-1alpha | 0.57868 | 0.131068 |
| E-Selectin | 1.899381 | 0.024678 | MIP-1gamma | 0.771899 | 0.4289 |
| Fibulin 3 | 0.919675 | 0.113811 | MIP2 | 3.491928 | 0.00588 |
| G-CSF | 0.916667 | 0.604652 | MMP-3 | 1.156293 | 0.075835 |
| GM-CSF | 0.25878 | 0.001208 | MMP-9 | 1.558735 | 0.097073 |
| Hepcidin | 2.166403 | 0.00372 | Mtor | 0.766871 | 0.153353 |
| HGF | 0.955707 | 0.693253 | Osteopontin | 2.449626 | 0.00128 |
| HMGB1 | 1.549026 | 0.015998 | Osteoprotegerin | 1.142857 | 0.788978 |
| ICAM-1 | 0.829701 | 0.094484 | Pentraxin3 | 0.561338 | 0.05006 |
| ICAM-5 | 0.826141 | 0.171191 | PF-4 | 1.386597 | 0.081717 |
| IFN-beta | 1.144144 | 0.746237 | progranulin | 5.394212 | 0.008483 |
| IFN-gamma | 1.873433 | 0.004392 | P-Selectin | 1.545129 | 0.00318 |
| IGFBP-1 | 1.82839 | 0.008875 | RAGE | 3.945578 | 0.000767 |
| IL-1alpha | 1.037397 | 0.890337 | RANTES | 1.439516 | 0.320478 |
| IL-1beta | 0.190476 | 0.041796 | SPARC | 0.893333 | 0.495881 |
| IL-1 Ra | 0.964664 | 0.906241 | TARC | 0.167296 | 0.060225 |
| IL-10 | 0.986836 | 0.949173 | TGF-beta1 | 0.805556 | 0.241746 |
| IL-11 | 0.965054 | 0.712353 | Tie-2 | 1.12428 | 0.787794 |
| IL-13 | 1.031125 | 0.853899 | TIMP-1 | 1.611777 | 0.003108 |
| IL-15 | 1.103203 | 0.878878 | TIMP-2 | 1.329993 | 0.053377 |
| IL-16 | 1.380966 | 0.043586 | TNF-alpha | 1.455331 | 0.00749 |
| IL-17 | 0.840708 | 0.727145 | TNF-beta | 1.13951 | 0.262844 |
| IL-17E | 2.876866 | 0.144598 | TRAIL | 2.216689 | 0.039016 |
| IL-17F | 0.258706 | 0.043138 | TREM-1 | 0.811765 | 0.735383 |
| IL-2 | 1.237805 | 0.33779 | TWEAK | 4.277027 | 0.007849 |
| IL-20 | 1.118825 | 0.756828 | VE-Cadherin | 1.569469 | 0.044583 |
| IL-21 | 0.899819 | 0.537521 | VEGF | 1.31441 | 0.700433 |
| IL-22 | 1.13549 | 0.221455 | VEGF-B | 1.424484 | 0.025681 |
| IL-23 | 1.041143 | 0.784776 | VEGF-C | 1.053023 | 0.391639 |
| IL-28 | 1.042165 | 0.728751 | VEGF-D | 1.725806 | 0.088402 |
