# Supplementary figures and images for: Hepcidin contributes to Swedish mutant APP-induced osteoclastogenesis and trabecular bone loss
Source: Bone Res. 2021 Jun 9;9:31. doi: 10.1038/s41413-021-00146-0 (PMC8190093; doi:10.1038/s41413-021-00146-0)

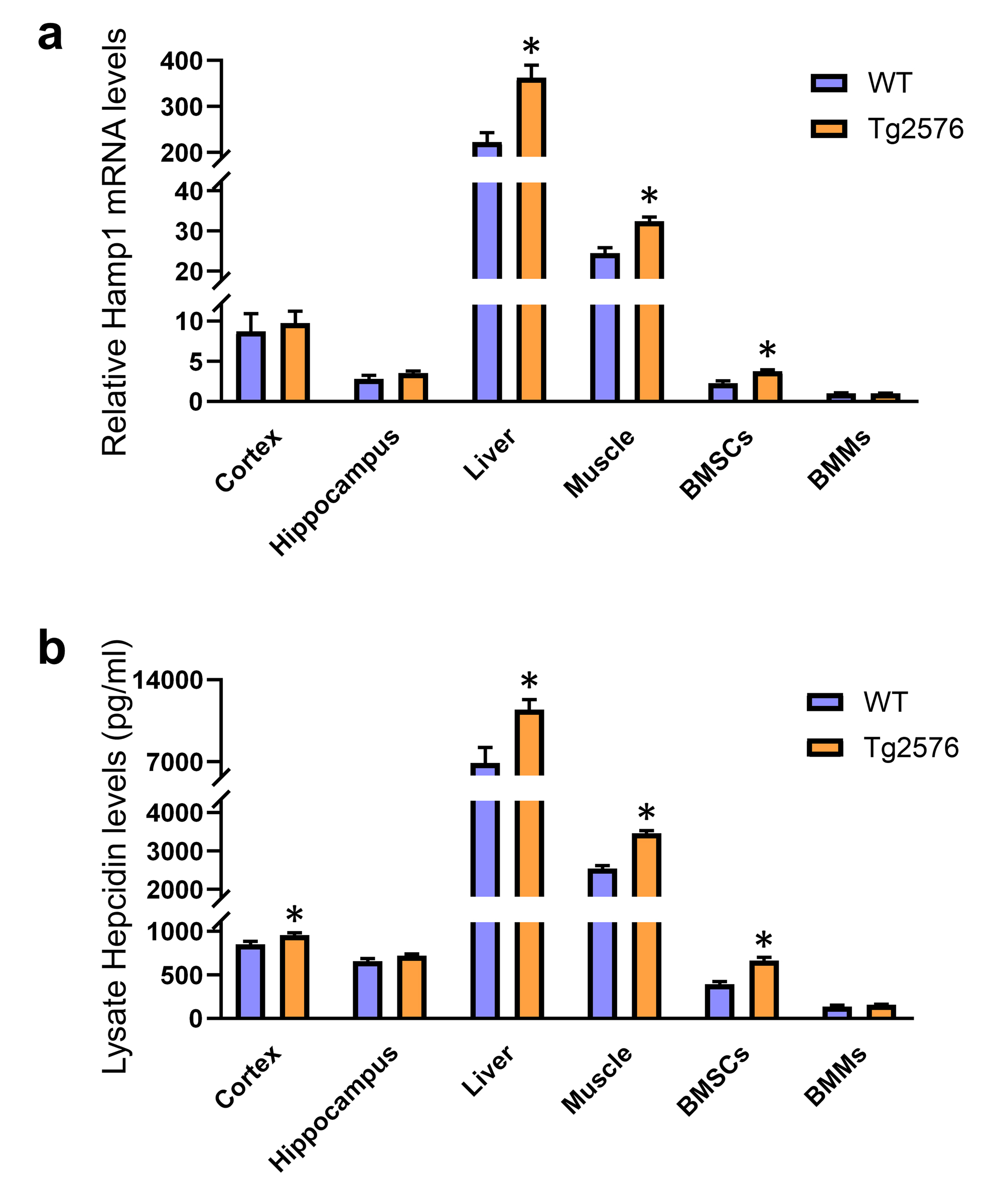

Supplement: Supplementary file 2 — Supplementary Fig S1 [file 41413_2021_146_MOESM2_ESM.jpg]

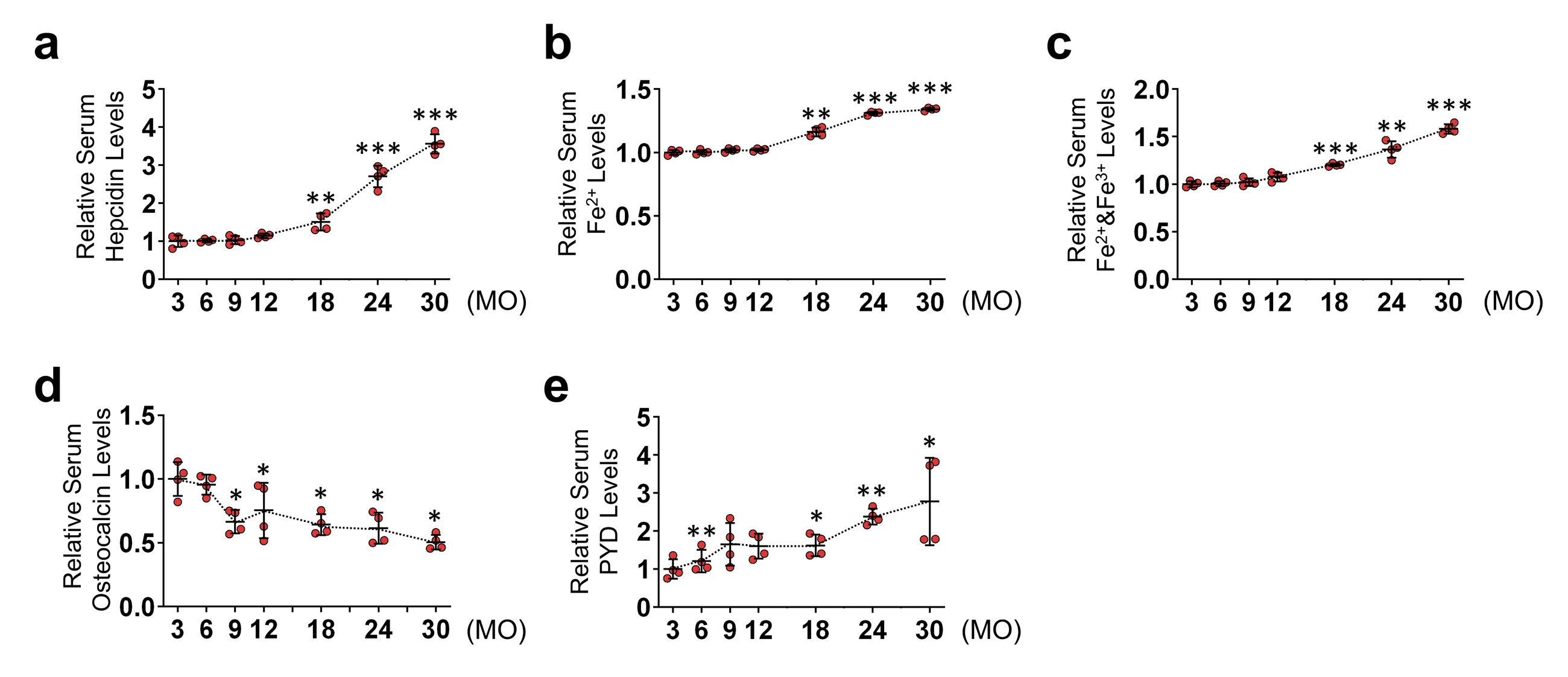

Supplement: Supplementary file 3 — Supplementary Fig S2 [file 41413_2021_146_MOESM3_ESM.jpg]

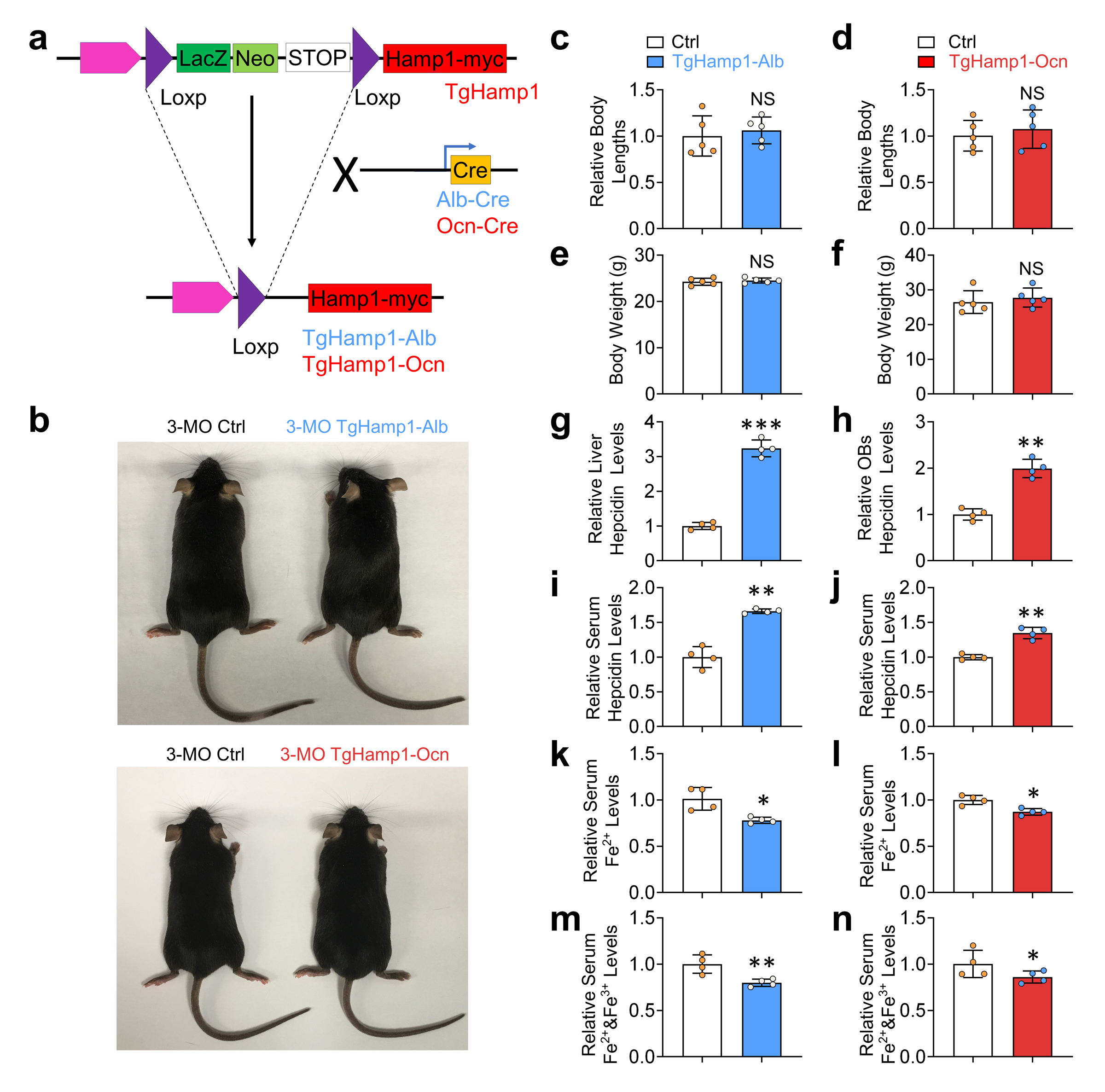

Supplement: Supplementary file 4 — Supplementary Fig S3 [file 41413_2021_146_MOESM4_ESM.jpg]

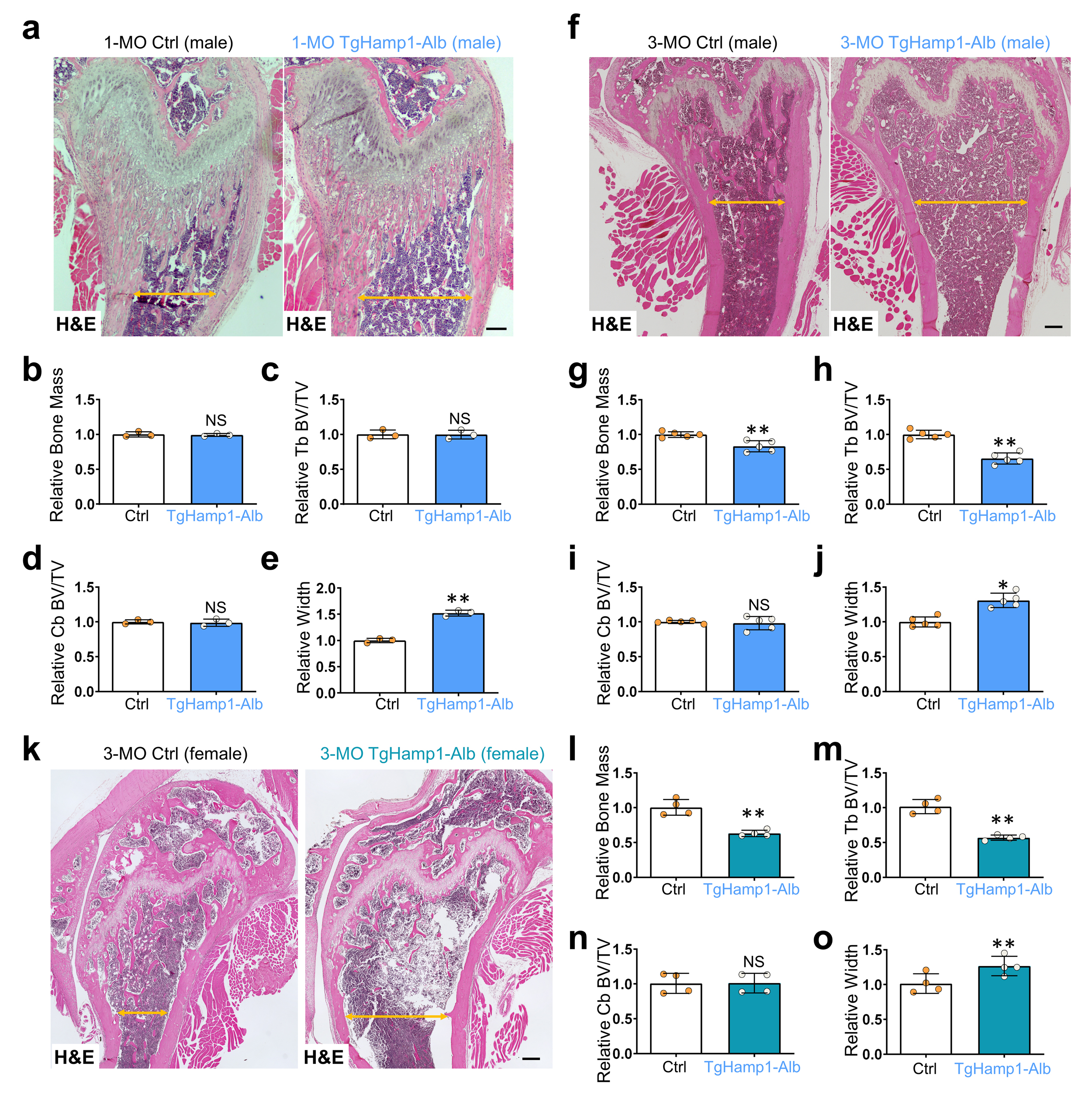

Supplement: Supplementary file 5 — Supplementary Fig S4 [file 41413_2021_146_MOESM5_ESM.jpg]

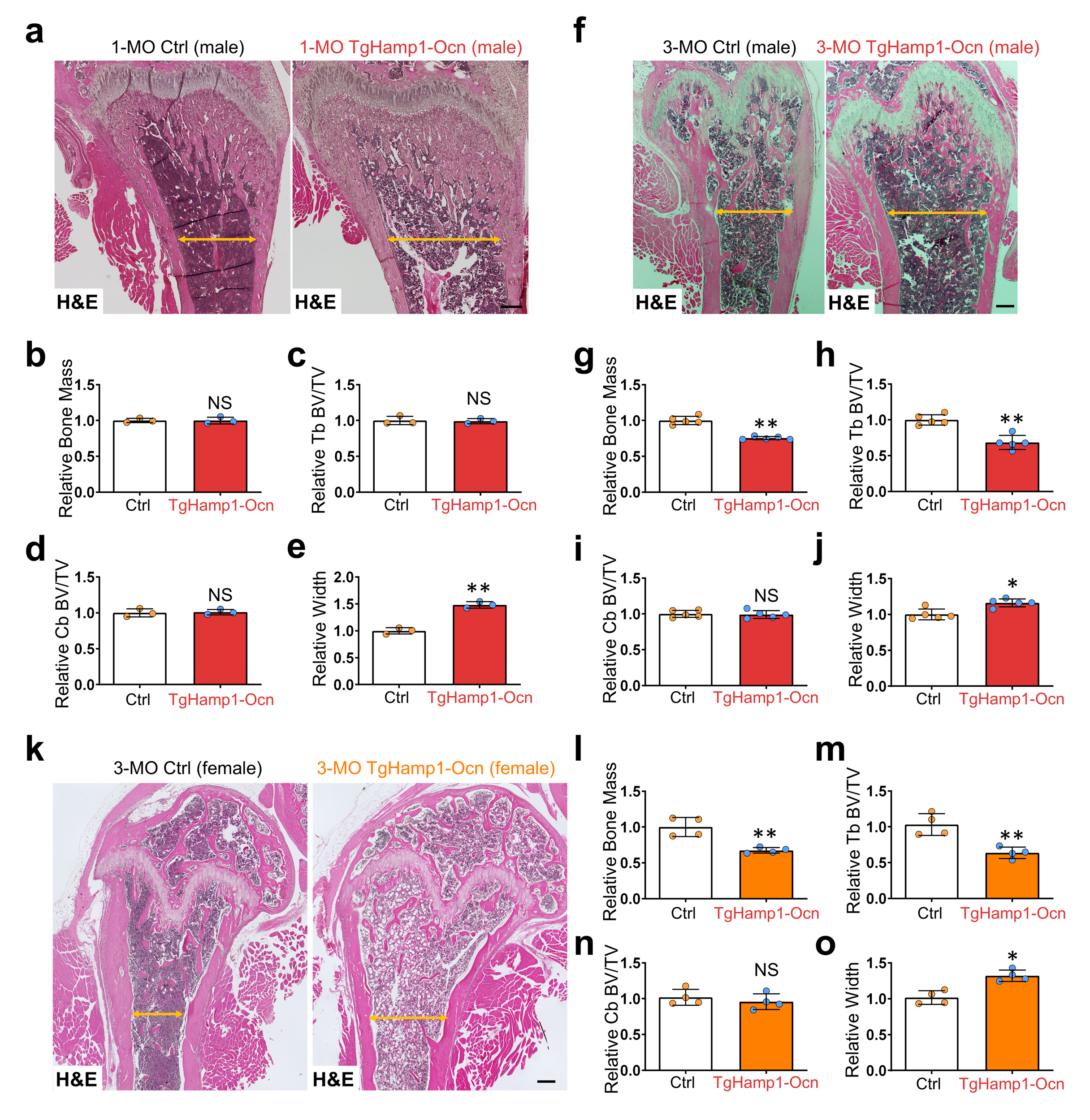

Supplement: Supplementary file 6 — Supplementary Fig S5 [file 41413_2021_146_MOESM6_ESM.jpg]

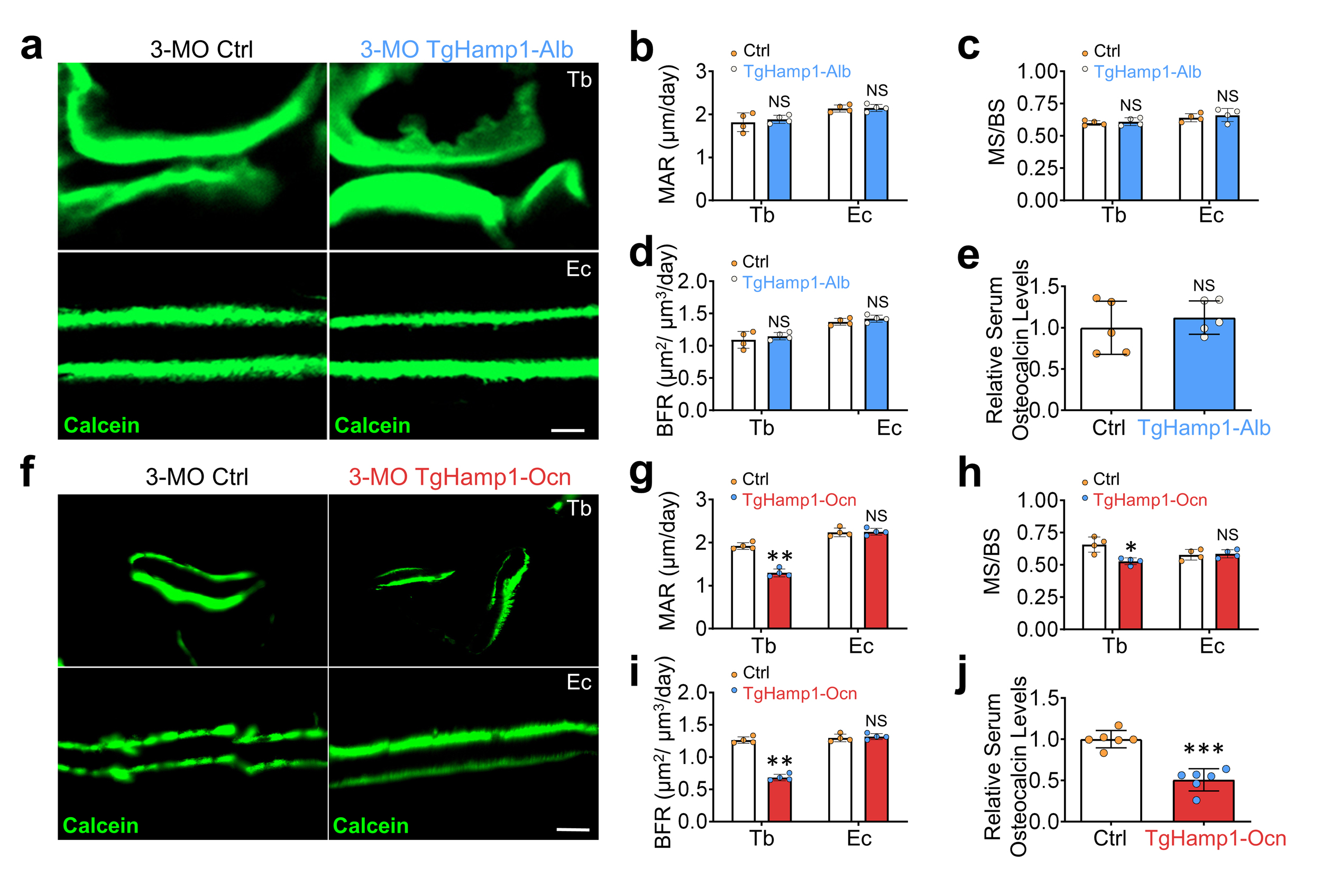

Supplement: Supplementary file 7 — Supplementary Fig S6 [file 41413_2021_146_MOESM7_ESM.jpg]

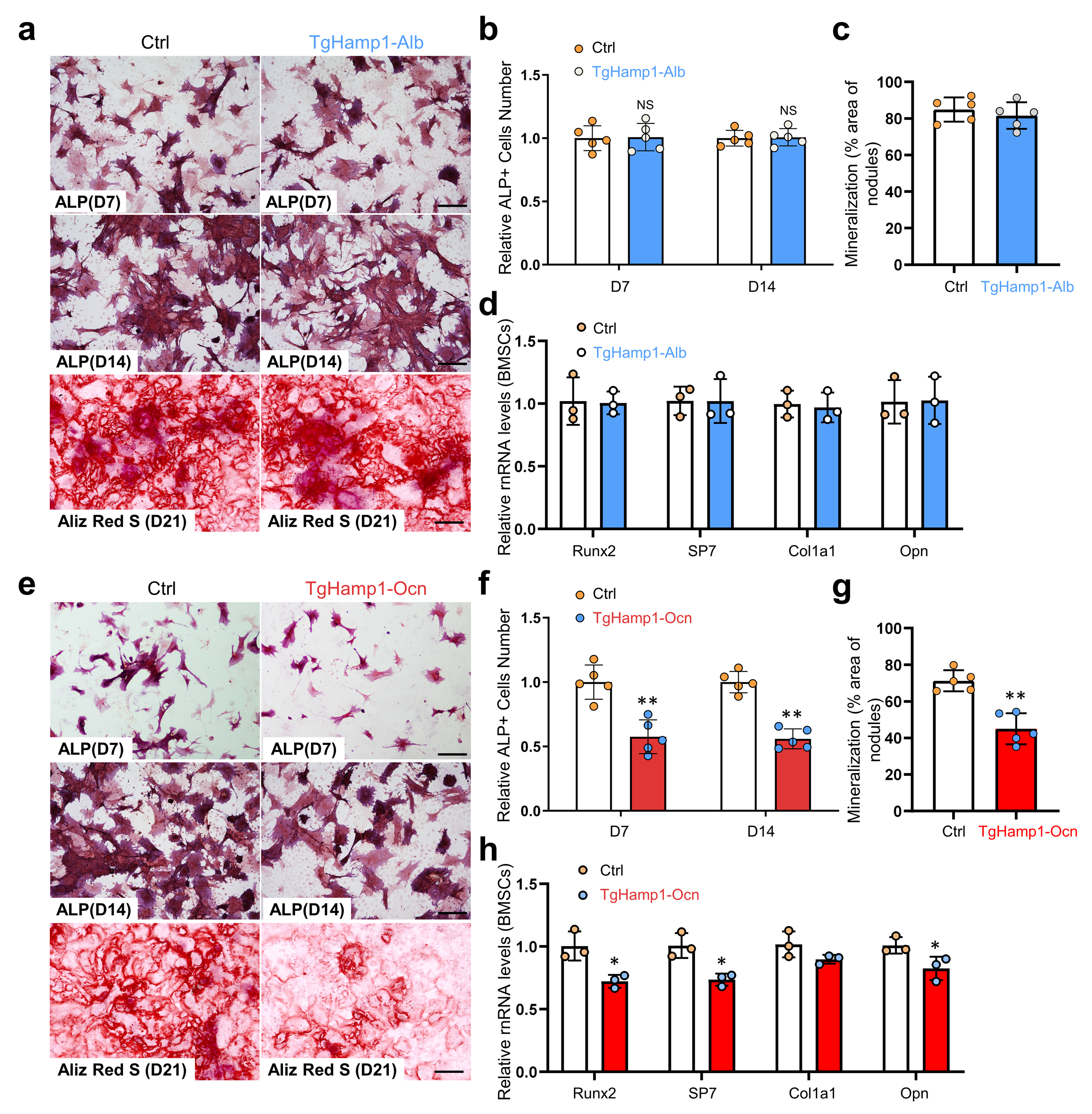

Supplement: Supplementary file 8 — Supplementary Fig S7 [file 41413_2021_146_MOESM8_ESM.jpg]

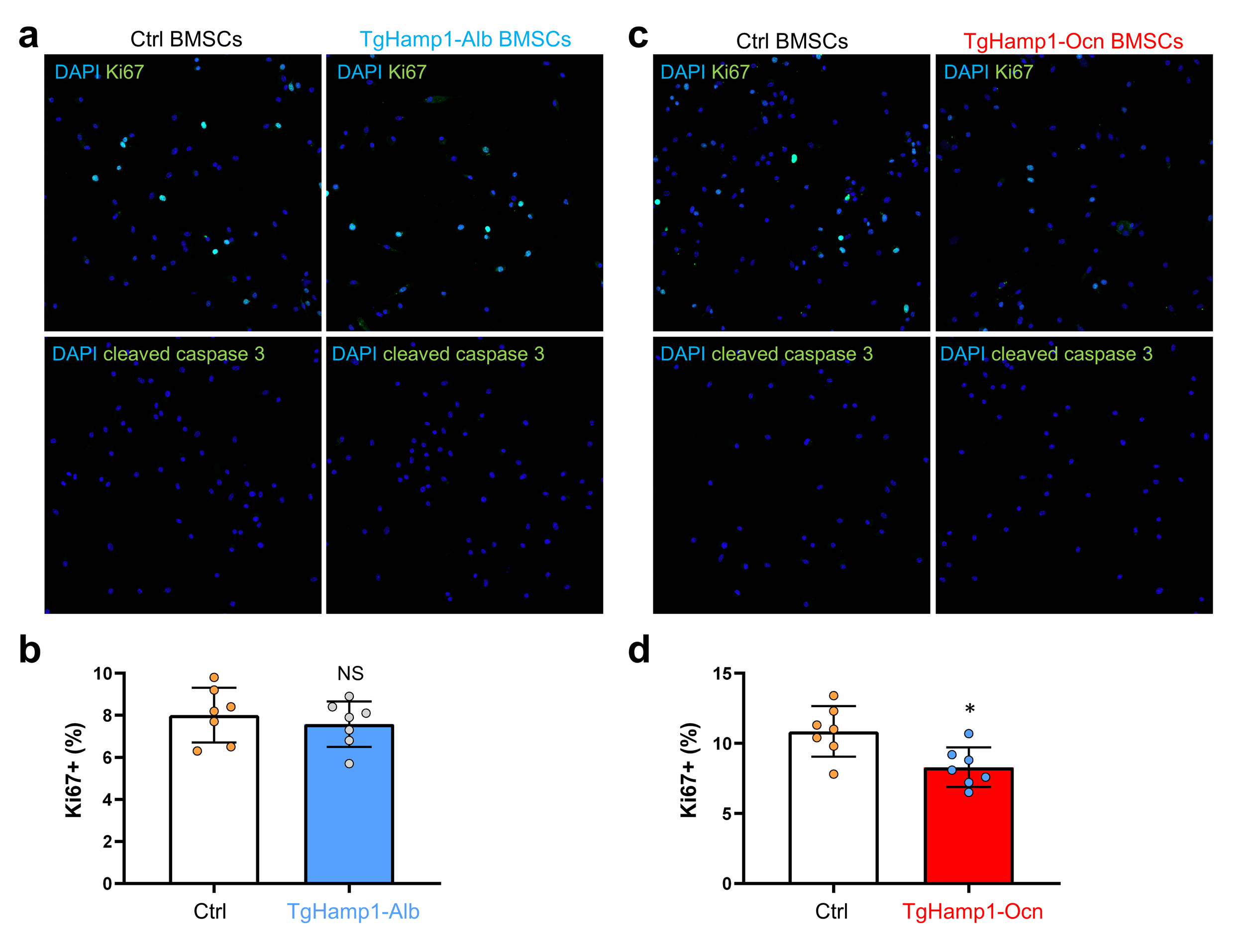

Supplement: Supplementary file 9 — Supplementary Fig S8 [file 41413_2021_146_MOESM9_ESM.jpg]

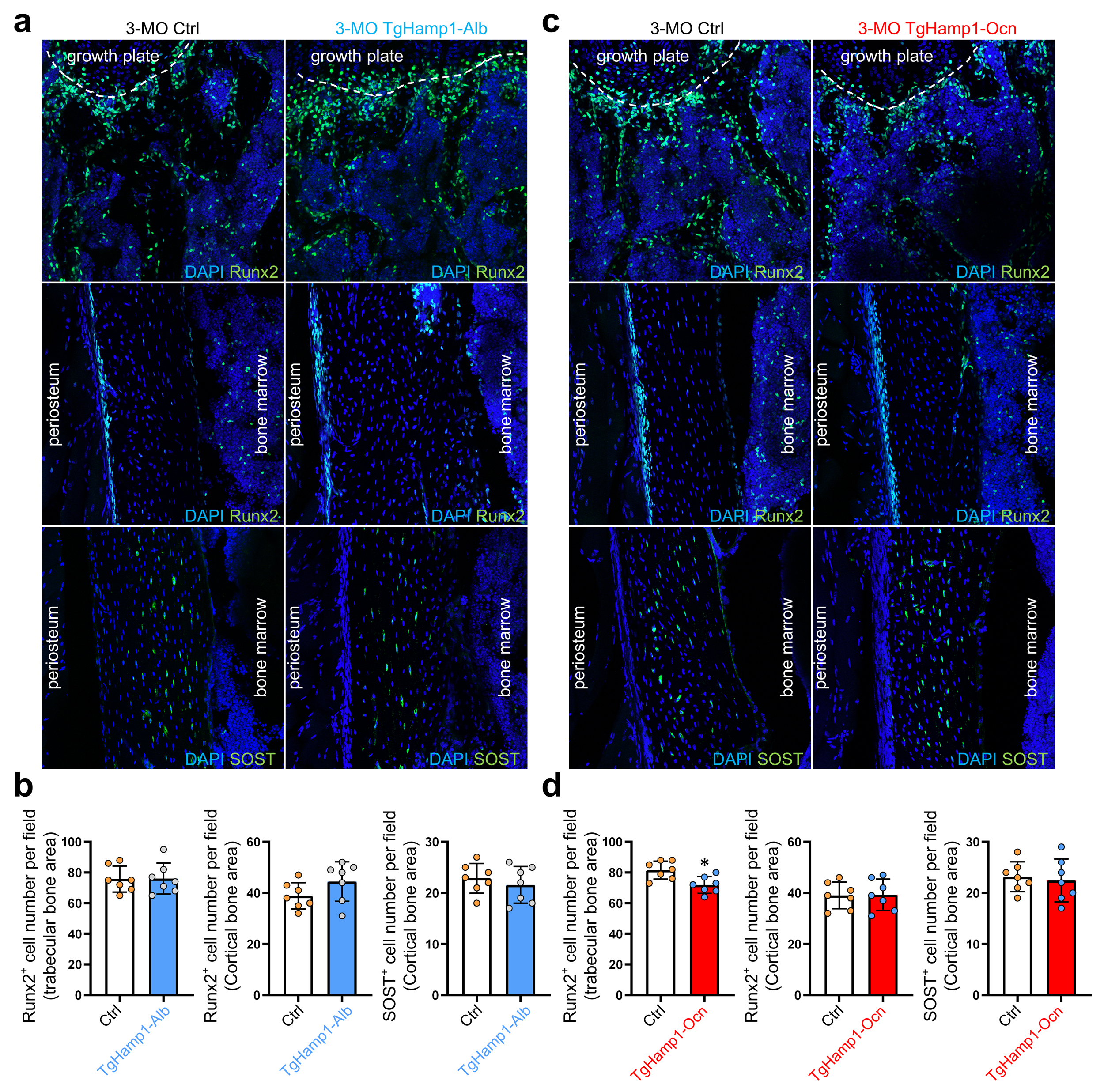

Supplement: Supplementary file 10 — Supplementary Fig S9 [file 41413_2021_146_MOESM10_ESM.jpg]

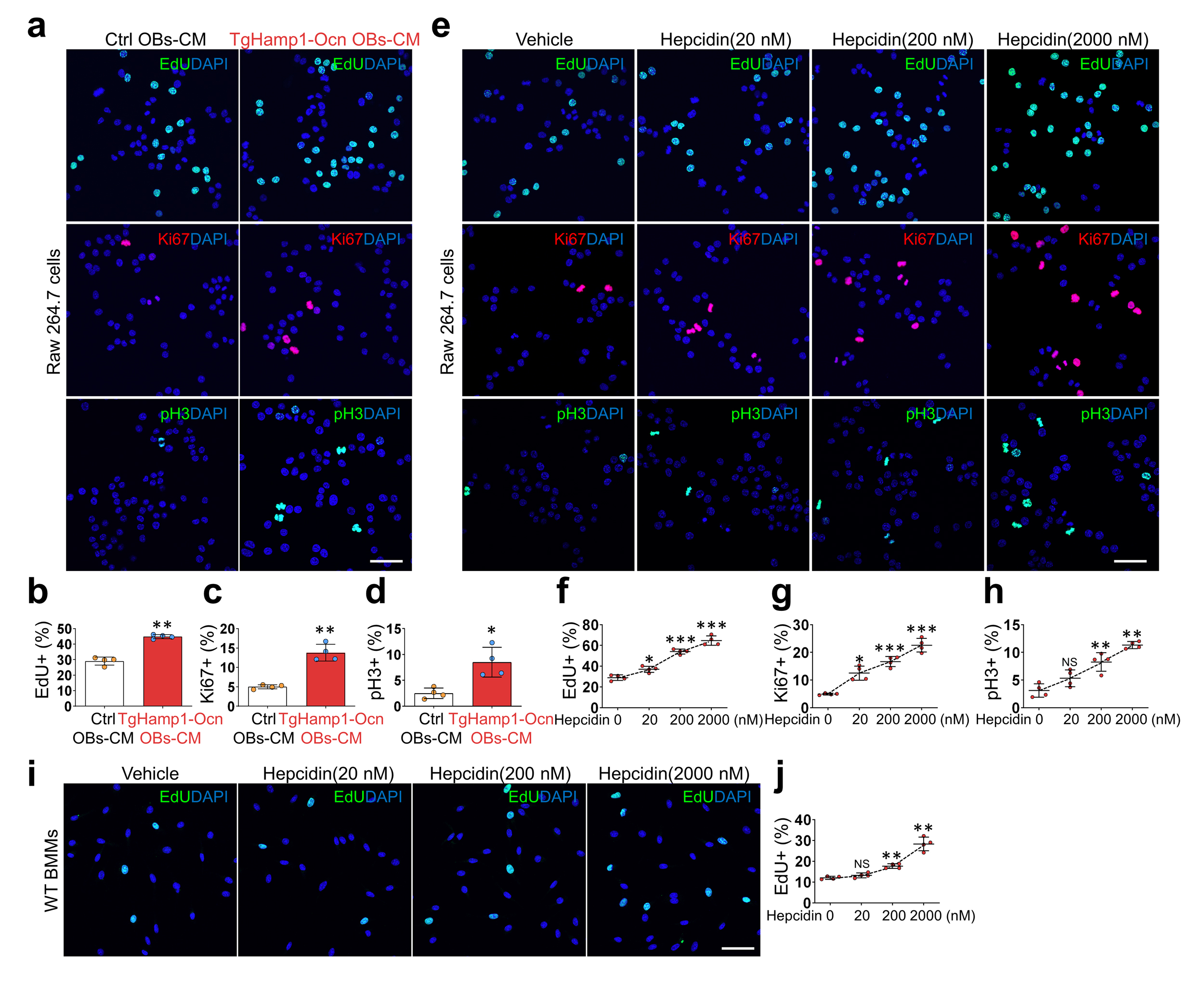

Supplement: Supplementary file 11 — Supplementary Fig S10 [file 41413_2021_146_MOESM11_ESM.jpg]

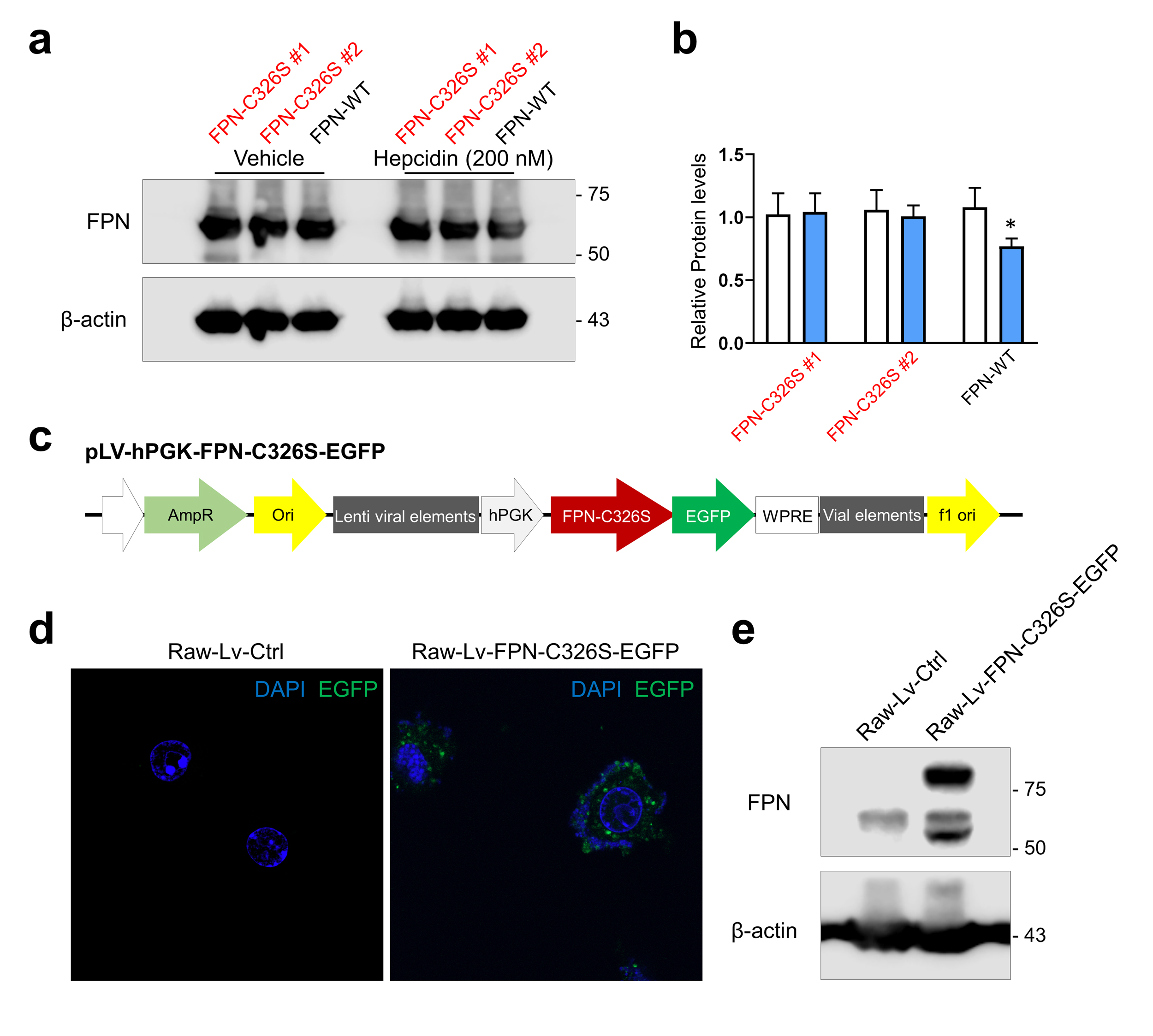

Supplement: Supplementary file 12 — Supplementary Fig S11 [file 41413_2021_146_MOESM12_ESM.jpg]

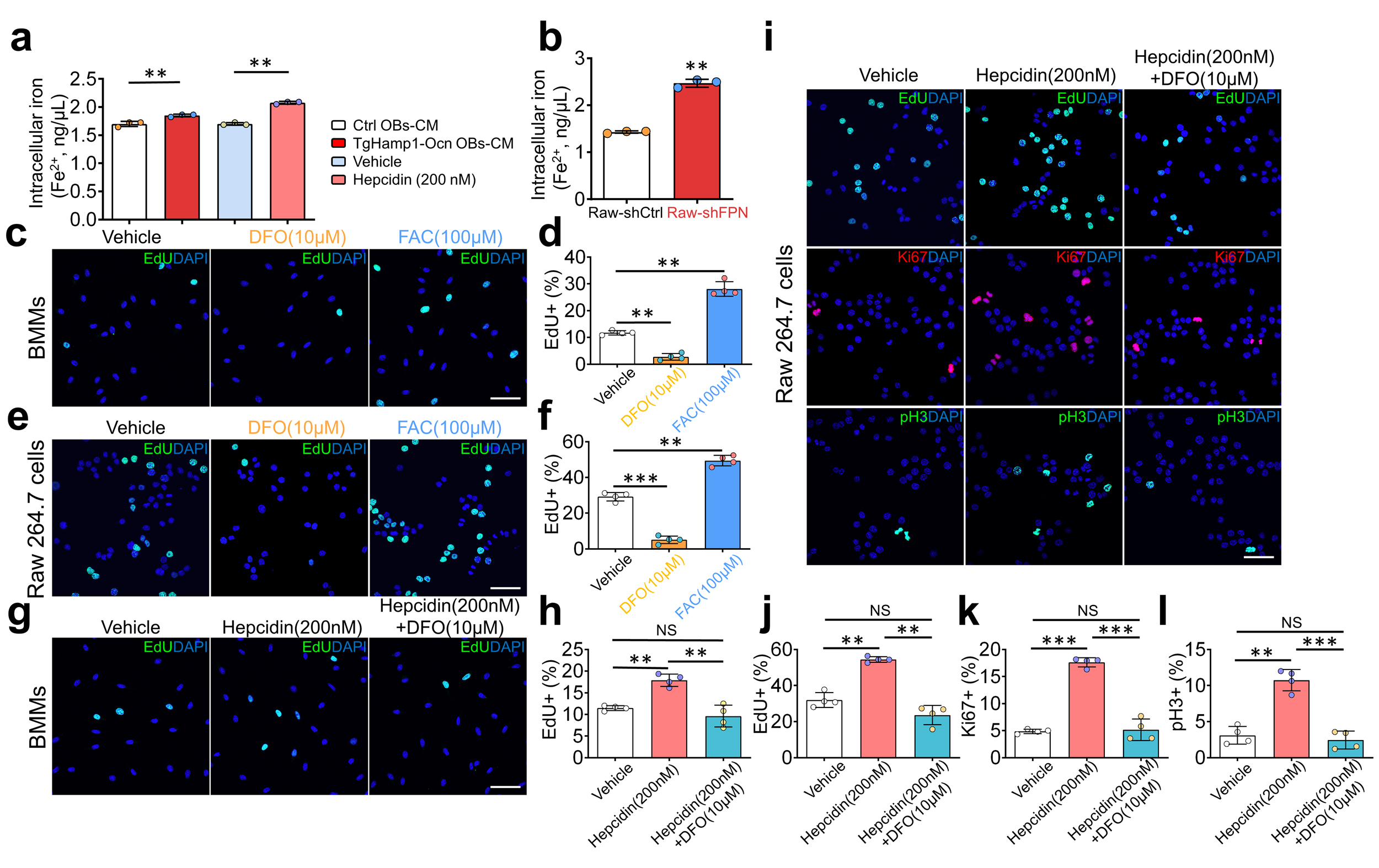

Supplement: Supplementary file 13 — Supplementary Fig S12 [file 41413_2021_146_MOESM13_ESM.jpg]

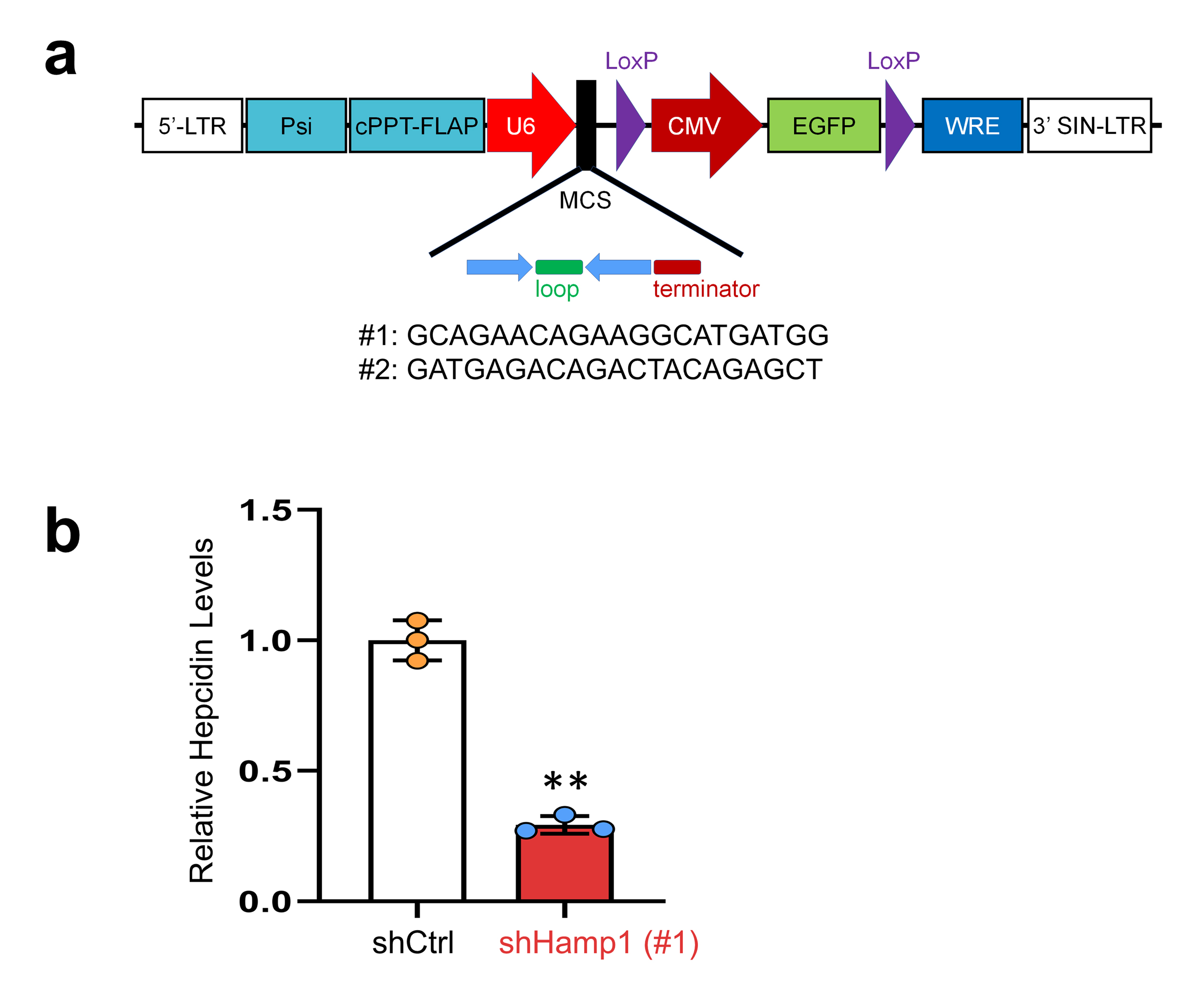

Supplement: Supplementary file 14 — Supplementary Fig S13 [file 41413_2021_146_MOESM14_ESM.jpg]
